# Dietary phosphate exposure–strategies to protect vulnerable population groups

**DOI:** 10.1007/s00204-025-04274-y

**Published:** 2026-02-07

**Authors:** Alfonso Lampen, Dirk W. Lachenmeier, Patrick Diel, Regina Ensenauer, Lara Frommherz, Sabine Guth, Hans-Ulrich Humpf, Sabine E. Kulling, María A. Villar-Fernández, Wim Wätjen, Angela Mally, Pablo Steinberg

**Affiliations:** 1 Institute for Food Quality and Food Safety, University of Veterinary Medicine Hannover, Bischofsholer Damm 15, 30173 Hannover, Germany; 2 Chemisches und Veterinäruntersuchungsamt Karlsruhe, Weißenburger Straße 3, 76187 Karlsruhe, Germany; 3 Department of Molecular and Cellular Sports Medicine, Institute of Cardiovascular Research and Sports Medicine, German Sport University Cologne, Am Sportpark Müngersdorf 6, 50933 Cologne, Germany; 4 Faculty of Medicine, Heinrich Heine University Düsseldorf, Moorenstraße 5, 40225 Düsseldorf, Germany; 5 76307 Karlsbad, Germany; 6 Leibniz Research Centre for Working Environment and Human Factors (IfADo), Ardeystr. 67, 44139 Dortmund, Germany; 7 Institute of Food Chemistry, Universität Münster, Corrensstraße 45, 48149 Münster, Germany; 8 Institute of Applied Biosciences, Karlsruhe Institute of Technology (KIT), 76131 Karlsruhe, Germany; 9 Institut für Agrar- und Ernährungswissenschaften, Martin-Luther-Universität Halle-Wittenberg, Weinbergweg 22, 06120 Halle (Saale), Germany; 10 Department of Toxicology, University of Würzburg, Versbacher Str. 9, 97078 Würzburg, Germany; 11 50968 Cologne, Germany

**Keywords:** Phosphates, Food additives, Risk assessment, Dietary exposure, Chronic kidney disease, Children

## Abstract

Phosphorus is commonly part of the diets in developed countries, both as a natural component of protein-rich foods and as a food additive. Consumption of food containing high amounts of phosphate as a food additive has continued to rise over time, resulting in increasing dietary exposure to phosphate. In 2019, an evaluation of phosphoric acid and phosphates conducted by the European Food Safety Authority (EFSA) identified young populations as groups with exposures exceeding the Acceptable Daily Intake (ADI), and also raised concern that the current ADI may not be sufficiently protective for individuals with an impaired renal function, which may account for 10% of the general population. The Senate Commission on Food Safety (SKLM) of the German Research Foundation (DFG) critically reviewed the safety of dietary phosphate, with a particular focus on the kidney as the primary target organ, taking into account the occurrence and content of phosphate in food, the most recent exposure estimates, the bioavailability of phosphate from different sources, the evidence linking excessive phosphate intake to kidney damage in animal models and humans as well as the exceedance of the current ADI in children. Moreover, the SKLM identified data gaps and research needs that should be addressed to improve the risk assessment of phosphate, with a special focus on vulnerable population groups. Based on the presented evidence, the Commission concludes that excessive dietary phosphate intake warrants further attention regarding possible health effects in vulnerable population groups or at exposure levels exceeding the ADI. Finally, the SKLM suggests a battery of risk management measures to reduce dietary exposure to phosphate, particularly in infants, toddlers and children, and to protect patients with chronic renal diseases.

**Supplementary Information:**

The online version contains supplementary material available at 10.1007/s00204-025-04274-y.

## Introduction

Phosphorus (element P) is essential for numerous physiological processes. It is commonly found in the diets of inhabitants of developed countries, both as a natural component of protein-rich foods and as a food additive in form of phosphoric acid (E338), phosphate salts (E339–341, E343, E450–451) and polyphosphate (E452). Foods that are rich in phosphate additives contain particularly high levels of inorganic phosphate. With the increasing consumption of such foods, dietary intake of phosphate additives continues to rise, most likely leading to a corresponding increase in the overall phosphate exposure (Ritz et al. 2012). Unlike naturally occurring phosphate in food, which is mainly present as organic phosphate esters requiring hydrolysis prior to absorption in the gastrointestinal tract, phosphate from food additives is readily bioavailable and almost completely absorbed in the gastrointestinal tract. However, interpreting data on phosphorus and phosphate levels in food is challenging, as some sources report total phosphorus levels, while others specify phosphate concentration. Phosphorus amounts can be converted to phosphate contents by multiplying by a factor of 3.0661; conversely, phosphate amounts can be converted to phosphorus concentrations by dividing by the same value (for conversion of various phosphates to phosphorous pentoxide (P_2_O_5_) or elemental phosphorous (P), refer to appendix B in (EFSA 2019)).

In the EU, phosphates are authorized food additives in accordance with Annex II and III of Regulation (EC) No 1333/2008. In 2019, the EFSA Panel on Food Additives and Flavourings added to Food (FAF) provided a scientific opinion re-evaluating the safety of phosphates (E338–341, E343, E450–452) as food additives (EFSA 2019). In this opinion, a group acceptable daily intake (ADI) for phosphates, expressed as phosphorus, of 40 mg/kg body weight (bw) per day was proposed, encompassing the phosphorus intake from natural sources and from food additives.

While the association between dietary phosphate intake and serum phosphate levels appears to be modest at best in healthy individuals, increased phosphate intake in patients with chronic kidney disease (CKD) has been linked to several adverse clinical outcomes, *e.g.,* increased cardiovascular risk and mortality (Go et al. 2004; Palmer et al. 2011; Rubio-Aliaga 2020; Rubio-Aliaga and Krapf 2022; Voormolen et al. 2007). Accordingly, EFSA concluded that the established ADI is only protective for healthy adults and does not apply to individuals with moderate to severe renal impairment. Given that approximately 10% of the population suffer from a moderate to severe reduction in renal function, this poses a potential public health concern, as a notable percentage of the population may not be covered by the established ADI. Thus, it should be critically discussed whether additional measures are needed to protect this vulnerable group.

Furthermore, the EFSA panel noted that in the estimated overall dietary exposure scenario, based on analytical data, exposure to phosphorus/phosphate exceeded the proposed ADI for infants^1^ (> 12 weeks to < 12 months), toddlers (≥ 12 to < 36 months) and other children^2^ (≥ 36 months to < 10 years) already at the mean level, and for infants, toddlers, children and adolescents (≥ 10 to < 18 years) at the 95th percentile. The EFSA panel also noted that dietary exposure to phosphates due to their use as food additives in food supplements exceeds the proposed ADI in children, adults (≥ 18 to < 65 years) and the elderly (≥ 65 years).

The EFSA risk assessment focused on the general healthy population but identified patients with chronic renal failure and young population groups (infants, toddlers and other children) – which are additionally exposed to phosphate levels exceeding the ADI – as potentially vulnerable groups. However, the assessment did not elaborate further on possible health effects. Based on concerns that dietary intake of phosphate may negatively impact the health of these vulnerable population groups, the Senate Commission on Food Safety (SKLM) of the German Research Foundation (DFG) critically reviewed the safety of dietary phosphate, based on currently available scientific data. These included phosphate levels and occurrence in foods, the most recent exposure assessments, the bioavailability of phosphorus from various dietary sources, and the health effects of phosphate – particularly focusing on kidney damage as the most sensitive endpoint used to establish the current ADI, as well as the observed exceedance of the ADI in children. The present evaluation builds upon and extends the 2019 EFSA opinion by focusing primarily on CKD and vulnerable subpopulation groups (infants, toddlers and other children), and includes new scientific data such as: I) recent studies (in animals and humans) on the correlation between dietary phosphorus/phosphate intake and phosphate levels in serum, II) updated evidence of kidney damage associated with excessive phosphorus/phosphate intake in both animal and human studies. In addition, new exposure data based on the German Total Diet Study (BfR-MEAL^3^ Study), focusing on the German population, were included in the assessment and compared with the EFSA exposure estimates. An updated market analysis of phosphate-labelled food products in Germany and the EU was performed using the Mintel’s Global New Products Database (GNPD) to identify key food categories/groups that contribute most to dietary phosphate intake. Furthermore, this assessment identifies current data gaps and research needs that should be addressed to improve risk assessment of phosphorus/phosphate – particularly in vulnerable populations. Finally, this paper suggests a targeted strategy to reduce the dietary exposure to phosphate in order to better protect individuals with impaired renal function as well as infants, toddlers and other children.

## Dietary reference values for phosphorus

### Adequate intake (AI)

The EFSA Panel on Dietetic Products, Nutrition and Allergies (NDA) established adequate intakes (AIs) for phosphorus in all population groups (EFSA 2015). For infants (7–11 months), an AI of 160 mg phosphorus/day was derived; for children and adolescents (1–17 years), recommended AIs ranged from 250–640 mg phosphorus/day, and for adults (≥ 18 years), the recommended AI was 550 mg phosphorus/day (Table 1). It was considered that the AI for adults should also apply to pregnant and lactating women. Dietary Reference Values (DRVs) for calcium were used as a basis for the derivation of the AI, considering a molar calcium to phosphorus ratio of 1.4:1 in the whole body (EFSA 2015).

**Table 1:** Dietary reference values (DRVs) and upper intake levels (ULs) for phosphorus proposed by different regulatory bodies

Age	Dietary reference values				Upper intake level (UL)		
	Adequate intake (AI) (mg/day) (EFSA 2015)	Adequate intake (AI) (mg/day) (NHMRC 2006)	D-A-CH intake recommendations (mg/day) (D-A-CH 2015)	Recommended dietary allowance (RDA) (mg/day) (NIH 2019)	EFSA (2005) (mg/day)	VKM (2017) (mg/day)	US NIH (2019) (mg/day)
0–6 months	–	100	–	–	–	–	–
7–11/12 months	160	–	–	275	–	–	–
1–3 years	250	–	–	460	–	–	3,000
4–10 years	440	–	–	500^a^	–	–	3,000^a^
11–17 years	640	–	1,250^f^	1,250^b^	–	–	4,000^b^
≥ 18 years	550	–	700^c^	700^d^	–	3,000	4,000^d^
70 + years	–	–	–	700	–	–	3,000^e^
Pregnancy < 18 years	640	–	1,250^f^	1,250^f^	–	–	3,500^f^
Pregnancy ≥ 18 years	550	–	800^g^	700^g^	–	–	3500^g^
Breastfeeding < 18 years	640	–	1,250^f^	1,250^f^	–	–	4,000^f^
Breastfeeding ≥ 18 years	550	–	900^g^	700^g^	–	–	4,000^g^

^a^ 4–8 years, ^b^ 9–18 years, ^c^ > 18 years, ^d^ 19–70 years, ^e^ ≥ 71 years, ^f^ < 19 years, ^g^ ≥ 19 years

For infants (0–6 months), the National Health and Medical Research Council of Australia and the New Zealand Ministry of Health established an AI of 100 mg phosphorus/day. To derive this AI, the average intake of breast milk (780 mL/day) was multiplied by the average phosphorus concentration in breast milk (124 mg/L) (EFSA 2015; NHMRC 2006).

The Nutrition Societies in Germany, Austria and Switzerland (*i.e.,* the D-A-CH region) derived an intake reference value of 1,250 mg phosphorus/day for adolescents between 15 and 18 years of age, 700 mg phosphorus/day for adults (> 18 years) and 800 and 900 mg phosphorus/day for pregnant and breastfeeding women, respectively (D-A-CH 2015).

The US National Institutes of Health (NIH) established a recommended dietary allowance (RDA) of 275 mg/day for infants (7–12 months), 460 mg/day for toddlers (1–3 years), 1,250 mg/day for children and adolescents (9–18 years) and 700 mg/day for adults (> 18 years) (NIH 2019).

### Tolerable upper intake level

The EFSA NDA Panel did not establish a tolerable upper intake level (UL) for phosphorus, as the available data was considered insufficient (EFSA 2005). For European countries, EFSA estimated a mean intake of phosphorus from food and supplements of 1,000–1,500 mg/day, with intakes ranging up to about 2,600 mg/day. It was noted that intake levels of phosphorus of up to 3,000 mg/day can be tolerated by healthy adults without adverse effects. However, mild gastrointestinal symptoms have been observed in some individuals when doses above 750 mg/day have been ingested via phosphorus-containing supplements.

For adults, the Norwegian Scientific Committee for Food Safety (VKM) proposed a total phosphorus intake of 3,000 mg/day as a provisional UL and 750 mg/day as UL for supplements (VKM 2017).

The NIH established an UL of 3,000 mg/day for toddlers and children (1–8 years) and 4,000 mg/day for children (9–13 years) (NIH 2019). An UL of 4,000 mg/day was set for adolescents and adults (14–70 years), while a lower UL of 3,000 mg/day was set for the older population (≥ 71 years) (NIH 2019). Table 1 provides an overview of dietary reference values and upper intake levels for phosphorus proposed by different regulatory bodies.

## Occurrence and content in food

### Phosphate from natural sources

In studies on phosphate levels in food, it must be carefully considered whether the reported values are calculated as phosphorus (P), phosphoric acid (H_3_PO_4_), phosphate (PO₄^3-^), or phosphorus pentoxide (P₂O₅), as these require different conversion factors (see annex B in (EFSA 2019)). As the conversion factor is often not known, this review typically states the terminology used by the original authors and does not provide re-calculations.

Phosphorus-containing compounds may occur either naturally (e.g., phytic acid) or due to the addition of phosphate-based additives (see below). Most of the total phosphorus content in foods is expected to be present in the form of phosphates (NIH 2019). Many studies on nutrient levels in food only report analytical levels for total phosphorus content and do not provide precise information on the chemical form.

Phytic acid is the major storage form of phosphorus in plants, comprising 1–5% weight in cereals, legumes, oil seeds and nuts, and represents 50–85% of total phosphorus in plants (Gupta et al. 2015).

**Fig. 1:**
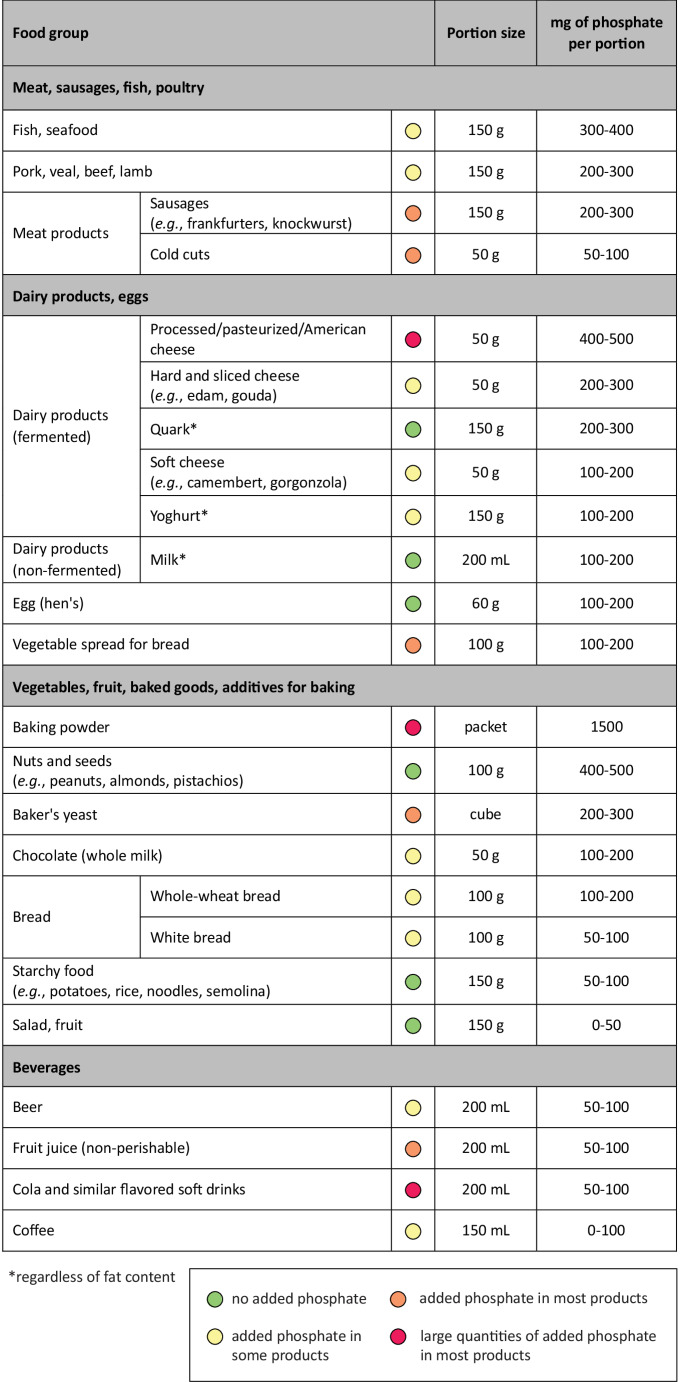
Phosphate content based on typical portion size across various food groups (modified from (Ritz et al. 2012)). Food groups are ordered according to phosphate content per portion. Coloured dots indicate the extent to which phosphate additives are present in products within each food group

The richest sources of dietary phosphate include dairy products (milk, cheese, yogurt), meat, poultry, fish, legumes (beans, peas, lentils), nuts, seeds and whole grains (Fig. 1). The phosphate concentrations per food portion typically range between 50 and 500 mg (Ritz et al. 2012).

**Fig. 2:**
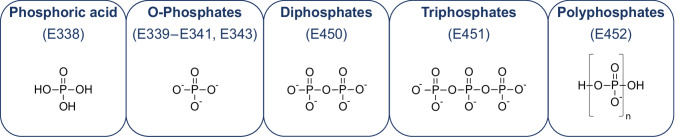
Phosphoric acid and phosphates used as food additives (modified from (Wieczorek et al. 2022))

**Fig. 3:**
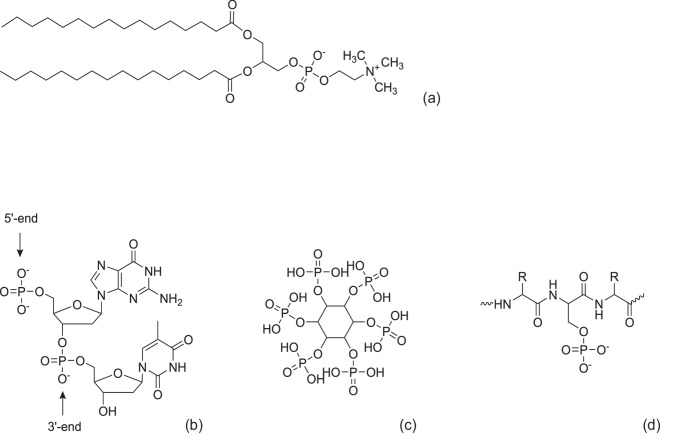
Selected molecular structures of organically bound phosphates **a** dipalmitoylphosphatidylcholine, **b** phosphate as component of a DNA strand, **c** phytic acid, **d** phosphoserine as part of a peptide chain

The different forms of phosphorus and species containing phosphorus that are present in foods include (Bump 2016):

- Inorganic phosphorus including phosphorus compounds (Fig. 2): This is the most common form of phosphorus in food and is found in both animal and plant foods.
- Organic phosphorus (Fig. 3): This form of phosphorus is found in plant foods and is bound to other molecules, such as phytic acid. Organic phosphorus is less easily absorbed by the body than inorganic phosphorus.

.

### Phosphate as a food additive

Phosphate additives are used in a wide variety of food products, including processed cheese, bread and baked goods, cereal-based foods, some alcoholic beverages, meat products, frozen meals, soft drinks, and candies (Calvo et al. 2023). Furthermore, new food groups, *e.g.,* plant-based meat alternatives, plant-based drinks (*e.g.,* soy, almond, oat drinks) as well as plant-based cheese and yoghurt alternatives can be a further source of added phosphates (Babich et al. 2025; Redan et al. 2023).

Phosphate additives are used in food for a variety of reasons, including improvement of the texture and flavor of food, increase of shelf-life, and prevention of discoloration (NIH 2019).

The food surveillance institutions regularly control the maximum levels for authorized phosphorus compounds, *e.g.,* phosphoric acid, phosphates and di-, tri- and polyphosphates (E338–452). The maximum levels refer to individual compounds or a combination of them and are expressed as P_2_O_5_ (EC 2008) (Electronic Supplementary Material Table S1). Therefore, there is no official control data available on the different phosphorus species (BVL 2021).

The regulation of phosphate addition to various food categories as specified in Regulation (EC) No 1333/2008 outlines a broad range of maximum levels for phosphates (E338–E341, E343, E450–452) (EC 2008), which include phosphoric acid and its sodium, potassium, calcium, and magnesium salts, as well as di-, tri-, and polyphosphates. For foods in dried powdered form, a general maximum level of 10,000 mg/kg or mg/L of phosphates applies, unless a different limit is explicitly stated or the food is specifically exempted (such as infant food).

Processed cheese, self-raising flour, certain bakery products and beverage whiteners have some of the highest permitted phosphate levels, with a maximum of 20,000 mg/kg for processed cheese, self-raising flour and certain bakery products and up to 50,000 mg/kg for beverage whiteners used in vending machines. Lower limits apply for foods to be consumed as such, *e.g.,* fruit preparations with up to 800 mg/kg, or flavored beverages with up to 700 mg/L. For a detailed overview, see Electronic Supplementary Material Table S1**.**

While empirical data are mostly lacking, it may be hypothesized that phosphate exposure increases with higher consumption of food products containing additives (Calvo et al. 2023). This hypothesis is supported by findings that processed foods more frequently contain phosphate additives compared to minimally processed counterparts. Notably, the prevalence of phosphate additives varies by food category, exceeding 50% in beverages and dairy products (Picard et al. 2023).

Furthermore, Carrigan et al. (Carrigan et al. 2014) demonstrated that replacing foods with low added phosphate with alternatives containing high amounts of added phosphate in the US diet may increase phosphorus intake by approximately 60%, equivalent to an additional 600 mg P/day. In European diets, such an increase in phosphorus exposure may be attributed to the higher permitted levels of phosphate additives in certain food categories, as listed in Regulation (EC) No 1333/2008 on food additives (Annex II, Part E–Approved food additives and conditions of use by food category).

For example, processed cheese can contain up to 20,000 mg/kg of phosphate additives, compared to 1,000 mg/kg in regular cheese (classified as a general dairy product). Similarly, processed potato products in the sense of Regulation (EC) No 1333/2008 such as French fries, frozen or chilled potato specialities and dehydrated potato products can contain up to 5,000 mg/kg of phosphate additives, whereas the addition of phosphate additives is not permitted in the case of unprocessed potatoes. Other comparisons include processed nuts (5,000 mg/kg) vs. unprocessed nuts (0 mg/kg, not approved), and liquid eggs (10,000 mg/kg) vs. fresh eggs (0 mg/kg, not approved).

Watanabe et al. (2016) compared the phosphate content of food products with a higher degree of processing with the values of the reference table (Food Composition Table–Support for Nutritional Decisions (Philippi 2013)) (Watanabe et al. 2016). In most food groups, the phosphate concentrations in food products with a higher degree of processing were significantly higher (1.5–8 times) than these reference values.

This supports the concern that higher consumption of food products of complex composition including additives may lead to an increased phosphate exposure.

### Occurrence of phosphate additives in food products (Summarized data from Mintel´s Global New Product Database)

The Mintel’s Global New Products Database^4^ (GNPD) is a commercially available comprehensive market research tool that provides information on various products and their ingredients, as labeled by the manufacturer. It records product launches of packaged goods worldwide and was used to extract information on the use of phosphate-containing additives and their derivatives (E338–E341, E343, E450–E452), based on the mandatory ingredient information on labels. This approach does not account for phosphates that are naturally present in other ingredients.

The database query was conducted for food and beverages for the period from November 2018 to November 2023, limited to the 24 EU member states currently available (see Electronic Supplementary Material Table S2). In an additional query, the search was further limited to the German market region.

A total of 362,889 products were identified in the EU market in the relevant subcategories^5^ (in Germany: 57,041 products), of which 27,485 (7.6%) contained phosphate additives according to their labeling (in Germany: 4,895 (8.6%)). In the following sub-categories, more than 50% of the products included phosphates (EU market):

- Evaporated milk (69%, n(total) = 52)
- Growing-up milk (1–4 years) (72%, n(total) = 298)
- Baby formula (6–12 months) (75%, n(total) = 298)
- Baby formula (0–6 months) (78%, n(total) = 322)

Other notable food categories frequently containing phosphates were creamers (42%, n(total) = 131), instant noodles (43%, n(total) = 729), cakes, pastries and sweet goods (43%, n(total) = 10,526) as well as sandwiches/wraps (44%, n(total) = 959).

In addition, Mintel provides information on various demographic claims of the products, which are given by the manufacturer on the labels. The search results were subsequently narrowed down to those claims for products intended to be consumed by babies and toddlers (0–4 years) and children (5–12 years). The summarized results of the various queries are displayed in Electronic Supplementary Material Table S2.

This restriction by claim increases the proportion of phosphate-containing products to 19% (n(total) = 6,576) in the EU market and to 27% (n(total) = 924), when limited to the products only available on the German market. This increase can mainly be attributed to the frequent use of phosphate additives in baby formula and growing-up milk.

The summarized results also provide an insight into which phosphate additives are used most frequently (see Electronic Supplementary Material Table S2); this use differs depending on the food sub-categories. Overall, diphosphates (E450) were most often used.

## Exposure

The exposure to phosphorus from natural food sources varies depending on the diet, with protein intake being a major influencing factor (D'Alessandro et al. 2015; Kalantar-Zadeh et al. 2010).

The exposure to phosphate from various food additives can also vary depending on the type and amount of food additives consumed and was estimated to make up between 6 and 30% of the total average intake of phosphorus in the EU (EFSA 2013).

According to exposure estimates by EFSA, the average total phosphorus intake in the EU ranged from 251 mg P/person per day for infants to 1,625 mg P/person per day for adults, and the high exposure (95th percentile) from 331 mg P/person per day for infants to 2,728 mg P/person per day for adults (EFSA, 2019). In the US, the estimated average daily phosphorus intake from foods is 1,237 mg for children and adolescents aged 2–19 years, and approximately 1,189 mg for women and 1,596 mg for men aged 20 years and older (NIH 2019).

**Fig. 4:**
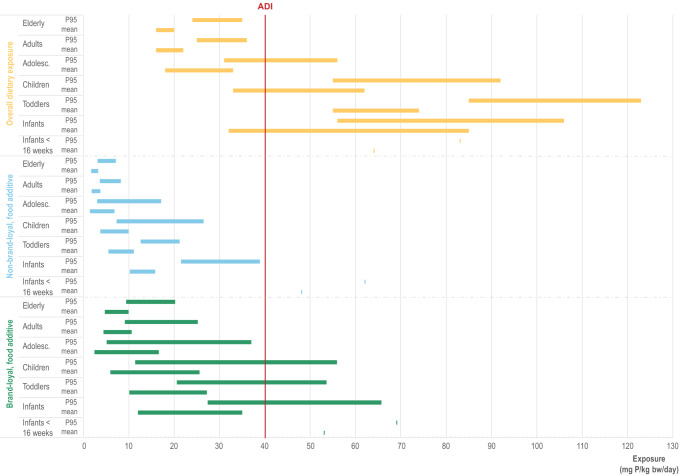
Dietary exposure to phosphorus (P) across seven population groups (minimum–maximum mean and 95^th^ percentile (P95) across the dietary surveys expressed in mg P/kg bw/day) as estimated by EFSA (2019). Three different exposure scenarios are shown: total phosphorus intake from the diet (yellow) and intake from the use of phosphate as food additive, considering a refined estimated exposure assessment scenario (non-brand-loyal (blue) and brand-loyal (green)). The red line indicates the acceptable daily intake (ADI) of 40 mg P/kg bw/day.

The dietary exposure to phosphate in seven population groups – infants (> 12 weeks to < 12 months), toddlers (≥ 12 months to < 36 months), other children (≥ 36 months to < 10 years), adolescents (≥ 10 years ≤ 17 years), adults (≥ 18 years < 65 years) and the elderly (≥ 65 years) – expressed in mg P/kg bw/day, as estimated by (EFSA 2019), is presented in Fig. 4. Three different exposure scenarios were considered: (i) total phosphorus intake from the diet, and intake from the use of phosphate as food additives (*e.g.,* E338–341, E343, E450–452), assuming either (ii) a non-brand-loyal scenario, or (iii) a brand-loyal scenario. The total phosphorus intake from the diet was estimated using analytical data provided by EU Member States, with the exception of those food categories for which no analytical data were available, where reported use levels were used. The analytical values reflect the total phosphorus content in food, regardless of its origin (naturally occurring or added as a food additive), and therefore provide a more accurate estimate of overall dietary phosphorus intake. To estimate phosphorus exposure exclusively from its use as food additives, a refined exposure assessment scenario was conducted based on use levels reported by the food industry (EFSA, 2019). This scenario includes only authorized uses for which such data were available to EFSA and assumes no phosphate content in food categories where no data were submitted. For the non-brand-loyal consumer scenario, exposure was estimated using the mean of the typical reported use levels across all authorized uses. In contrast, the brand-loyal consumer scenario assumes that a consumer is chronically exposed to phosphates at the maximum reported use level for the main contributing food category at the individual level, while the mean of typical reported use levels is taken into account for the remaining authorised applications of phosphate. EFSA considered the non-brand-loyal scenario as the most realistic long-term exposure scenario, but also concluded that, even in the refined exposure scenarios, uncertainties would likely lead to an overestimation of phosphate exposure (EFSA, 2019). The brand-loyal exposure scenario also might overestimate the intake from phosphate use as a food additive, but it cannot be excluded that this scenario might cover a subset of high-level consumers.

In the scenario based on analytical data representing the overall exposure to phosphorus from the diet (Fig. 4, yellow bars), the exposure exceeds the ADI of 40 mg/kg bw/day in infants, toddlers, and other children, both at the mean and high exposure levels. In adolescents, the ADI is exceeded at the 95th percentile of exposure. The maximum dietary exposure to phosphates from their use as food additives in infants, toddlers, and other children is close to the ADI at the mean consumption level and exceeds it at the 95th percentile in the brand-loyal scenario, while exposure levels in the non-brand-loyal scenario remain below the ADI (Fig. 4).

An overview of the different exposure scenarios to phosphate (all dietary sources, as a food additive: non-brand loyal, brand-loyal) in infants, toddlers and other children is presented in Electronic Supplementary Material S3. The consumption of food supplements and foods for special medical purposes (FSMP) are not considered in the three exposure scenarios described above. EFSA covered these exposure sources in two additional scenarios shown in Electronic Supplementary Material S4: (a) dietary exposure to phosphates (overall dietary exposure and exposure from their use as food additives) for food supplement consumers only, in children, adolescents, adults and the elderly, and (b) from their use in foods for FSMP in infants and toddlers (consumers only), as estimated by EFSA (2019). Of note, EFSA also estimated the phosphate exposure using maximum permitted levels (MPLs), referred to as “regulatory maximum level exposure assessment scenario” and established by EU regulation (Annex II to Regulation (EC) No 1333/2008). This scenario assumes that all relevant food categories contain phosphate at the maximum allowed concentration, which tends to overestimate actual consumer exposure, and is thus considered a conservative approach. Therefore, this scenario was not included in the present assessment, as the information on actual use levels provides more realistic exposure estimates.

In a recent study, the German Federal Institute for Risk Assessment (BfR) estimated the intake of phosphorus through food for different age groups (Ptok et al. 2024a, 2024b). The data from three consumption studies (KiESEL^6^ (0.5–5 years), EsKiMo II^7^ (6–11 years), and NVS II^8^ (14–80 years)) were combined with the data on the average phosphorus content of foods from the first German Total Diet Study (BfR-MEAL Study). The BfR-MEAL Study examined foods that cover more than 90% of those consumed in Germany. The total amount consumed over all days was calculated, then divided by the number of days that food was consumed and, if applicable, by the body weight of the study participants. Levels below the detection or quantification limit were set to zero or the value of the detection limit, respectively, using the modified lower bound approach (Ptok et al. 2024a, 2024b). It was estimated that 94% of adults, 91% of children and adolescents, and 98% of infants and young children meet the EFSA recommended adequate intake for the different age groups (EFSA 2015). In the age groups considered, except infants, the largest proportion of phosphorus intake (in total > 60%) comes from the food groups ‘cereals and cereal products’ (27–30%), ‘milk and dairy products’ (22–31%), and ‘meat and meat products’ (8–16%) (Ptok et al. 2024a, 2024b).

**Fig. 5:**
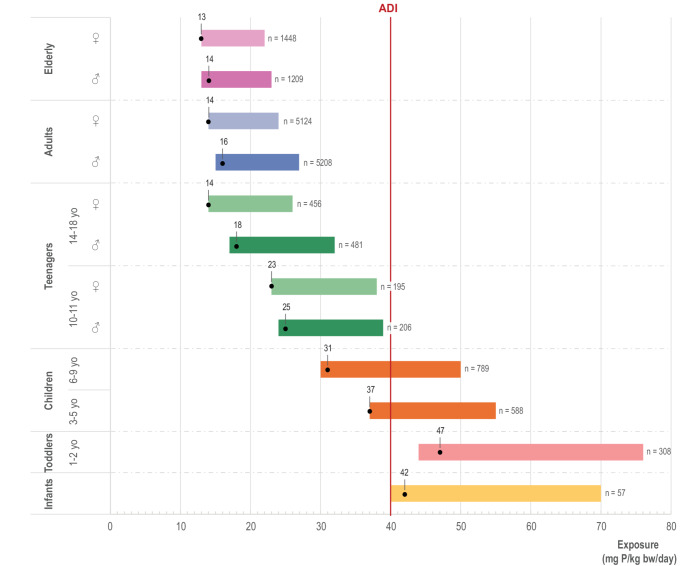
Dietary exposure to phosphorus (P) across six population groups in Germany (percentile 50 (P50) – percentile 95 (P95), modified lower bound in mg P/kg bw/day) as estimated by the BfR-MEAL study (2024). Black dots represent the mean values. The red line indicates the acceptable daily intake (ADI) of 40 mg P/kg bw/day. Source: Ptok et al. 2024a, 2024b

The intake of phosphorus from predominantly conventionally produced foods, expressed in mg/kg bw/day and in absolute mg/day, shows significant differences between the different age groups (Ptok et al. 2024a, 2024b) (Fig. 5). In up to two-year-old children, the average intake was about 42–47 mg/kg bw/day, with a peak (95th percentile) of up to 76 mg/kg bw/day. Children aged 6–9 years showed an average intake of 31 mg/kg bw/day, with the 95th percentile reaching up to 50 mg/kg bw/day. Adolescents aged 10–11 years showed intake averages of 23–25 mg/kg bw/day and a 95th percentile of up to 39 mg/kg bw/day. For adults, the average intake was 14–16 mg/kg bw/day, with a 95th percentile of up to 27 mg/kg bw/day.

Measured in absolute mg phosphorus per day (Ptok et al. 2024a, 2024b), infants (7–11 months old) consumed an average of 374 mg/day and 625 mg/day at the 95th percentile, exceeding the AI for this age group of 160 mg/day proposed by EFSA (EFSA 2015). Children aged 1–3 years and 4–5 years showed an average intake of 589 and 696 mg/day, with a 95th percentile of 891 and 1,018 mg/day, respectively. This compares to AI values recommended by EFSA of 250 and 440 mg/day. The group aged 6–10 years had an average intake of 834 mg/day and a 95th percentile of 1,271 mg/day, which exceeds the AI for this age group of 440 mg/day (EFSA 2015). Adolescents aged 14–17 years consumed an average of 1,201 mg/day for males and 840 mg/day for females, with the 95th percentile reaching up to 2,095 mg/day. The EFSA’s AI for this age group is 640 mg/day. For adults aged 18–80 years, the average intake is 1,272 mg/day for males and 942 mg/day for females, with a maximum 95th percentile of 2,111 mg/day, compared to the EFSA AI for adults of 550 mg/day (EFSA 2015). Considering the large standard deviations, these new German estimates are comparable to the data previously published by EFSA (2019), while the average and high exposure levels in Germany were typically 300–600 mg/day lower than the EFSA estimates.

Phosphorus intake tended to be higher among children and adolescents when compared to adults and infants. Moreover, the actual intake levels in most age groups significantly exceed the values recommended by EFSA (from 160 mg/day for infants up to 640 mg/day for adolescents). The prevalence of inadequate phosphorus intake levels in each of the considered population groups was very low (Ptok et al. 2024a, 2024b). Gender-specific differences in intake were particularly noticeable among adolescents and adults, with males generally exhibiting higher intake levels.

Children may be more exposed to phosphates from food and food additives than adults for several reasons:

- Children show a proportionally higher food consumption relative to their body weight. This also means that they consume relatively more nutrients, including phosphates.
- The digestive system of children continues to develop during the first years of life. In the course of this development, children may absorb more phosphates from food than adults. Furthermore, there may be an increased vulnerability of the growing and developing organism to adverse effects of toxicants.
- Children are more likely to be fed or eat food products containing high amounts of phosphate additives, *e.g.*, formulae, cereal-based foods, bread and rolls, fine bakery wares, processed cheese, meat products, sugars and syrups (EFSA 2019).

According to EFSA calculations, the exposure via infant formula or FSMP for high level consumers was estimated to range from 44–175 mg/kg bw/day total phosphorus based on the minimum and maximum levels for total phosphorus in infant formula according to the regulations^9^, regardless of the origin of the phosphorus in the formula (nutrient or food additive) (EFSA 2019).

Dietary exposure to phosphates from their use as food additives in FSMP in infants and toddlers (consumers only), as estimated by EFSA (2019), is presented in Electronic Supplementary Material S4 (B). Based on the regulatory maximum level exposure assessment scenario, estimated levels for infants (< 16 weeks), were 87 and 113 mg P/kg bw/day at the mean and 95th percentile, respectively. Although ADIs do not apply to infants under 16 weeks without further considerations (EFSA 2017), because of their distinct physiology with different metabolic and nutritional needs, these estimates are considerably above the general ADI. However, EFSA concluded that “the available data did not give rise to safety concerns in infants below 16 weeks of age consuming formula and food for medical purposes” (EFSA 2019). It was noted that “given the limits set by existing regulation, it seems not appropriate to use the ADI set for food additives only for infants formula, nor is it necessary to derive a numerical ADI applicable for this age group” (EFSA 2019). Therefore, the age group of infants < 16 weeks is not discussed further in the present paper. In the refined estimated exposure scenario, the mean exposure in infants (> 12 weeks to < 12 months) was 13–29 mg phosphorus/kg bw/day, which is below the ADI. However, at the maximum 95th percentile the estimated exposure was above the ADI (76 mg P/kg body weight/day). The estimated exposure for toddlers (≥ 12 to < 36 months) was below the ADI both at the mean and at the 95th percentile exposure.

The mean phosphorus concentration in human breast milk was reported to range between 110 and 160 mg/L, depending on the post-partum day (EFSA 2015; Gidrewicz and Fenton 2014). Based on a mean breast milk intake of 780 mL/day for infants (0–6 months) and an average phosphorus concentration in human breast milk of 124 mg/L, an AI of 100 mg P/day was derived (EFSA 2015; NHMRC 2006). Considering the highest reported mean phosphorus concentrations in breast milk (160 mg/L) and a breast milk intake of 0.839 kg/day at the 95th percentile (mean 0.778 kg/day; 95% CI = 0.717, 0.839; (da Costa et al. 2010)), corresponding to 0.815 L/day^10^, the maximum estimated phosphorus intake for exclusively breastfed infants is approximately 130 mg P/day (equivalent to 32.5 mg/kg bw/day for a bodyweight of 4 kg). This is considerably lower than the estimated intake from infant formula.

## Bioavailability of phosphorus from different food and sources

Phosphorus in food exists either as organic phosphorus, bound to proteins, lipids, and other molecules like inositol, or as inorganic phosphate. Inorganic phosphate, as found in food additives, is considered highly bioavailable, with absorption rates ranging from 80% to nearly 100% (Bell et al. 1977; Calvo et al. 2014; Cupisti and Kalantar-Zadeh 2013; EFSA 2019; Karp et al. 2012; St-Jules et al. 2017). This is explained by the fact that inorganic phosphate, typically in the form of phosphate salts, is water-soluble and readily dissolves in the digestive tract, allowing for efficient absorption without the need for enzymatic hydrolysis. Absorption mainly takes place in the jejunum of the small intestine through specific transporters (Cupisti and Kalantar-Zadeh 2013; EFSA 2019).

In contrast, the bioavailability of phosphorus from organic sources has been reported to range between 40 and 60%, with phosphorus from animal-based origin being more readily absorbed than plant-based phosphorus (Calvo et al. 2014). Phosphorus in animal-derived foods, such as meat and dairy products, is primarily present as organic phosphates within cells (Kalantar-Zadeh et al. 2010). These organic phosphates must be hydrolyzed by digestive enzymes. Typically, up to 60–70% of phosphorus from animal-based foods is absorbed (Calvo et al. 2014; Cupisti and Kalantar-Zadeh 2013; Kalantar-Zadeh et al. 2010; NIH 2019). The phosphorus in dairy products is particularly bioavailable, most likely due to the nature of its binding to proteins, which makes it more accessible during digestion.

Plant-based phosphorus, however, is less readily absorbed, with a bioavailability generally below 50% (EFSA 2019; Kalantar-Zadeh et al. 2010; Schlemmer et al. 2009). This reduced absorption is primarily due to the presence of phytate (myo-inositol hexakisphosphate), a phosphorus storage molecule in plants, especially present in beans, peas, cereals, and nuts.

Phytates bind phosphorus in the form of phosphate esters, which are resistant to hydrolysis by human digestive enzymes, due to the lack of phytase – the enzyme required for phosphorus release (EFSA 2019). As a result, phosphorus from phytate-rich foods is poorly absorbed. Some estimates suggest that the bioavailability of phosphate from phytates is limited (20–30%) and 40–50% of plant phosphorus is excreted without being absorbed (EFSA 2019). However, the content and bioavailability of phosphorus from grain and legume products are variable and depend on food preparation and processing. For instance, boiling and soaking can reduce phytate levels, and the leavening of bread with phytase-producing yeasts increases the bioavailability of phosphorus (EFSA 2015; Heaney 2012).

In summary, estimates based on digestibility suggest that the bioavailability of phosphorus increases from plant-based foods to animal-based foods, with the highest bioavailability found in the case of food additives.

However, St-Jules et al. (2017) reported that digestibility may not fully reflect bioavailability *in vivo* and overlooks interactions within the human digestive tract, as well as the effects of other nutrients on phosphorus metabolism (St-Jules et al. 2017). Based on 24-h urinary phosphorus measurements, phosphorus from food additives, although highly digestible, appears to be incompletely absorbed. Absorption may be influenced by various factors, such as the presence of other minerals in the intestinal lumen, *e.g.,* calcium, which can prevent absorption in the digestive tract by binding to phosphorus (Heaney 2012; O'Brien et al. 2014).

## Correlation between phosphate intake and phosphate levels in serum

### Evidence from human studies

Serum phosphate levels are regulated by a complex network involving primarily three key hormones, *i.e.,* parathyroid hormone (PTH), fibroblast growth factor 23 (FGF23) and calcitriol (1,25(OH)_2_D). These hormones coordinately maintain phosphate homeostasis by controlling intestinal phosphate absorption, reabsorption and excretion by the kidneys, as well as the flux between extracellular and skeletal phosphate pools. In healthy individuals, these homeostatic mechanisms, particularly rapid elimination of phosphate via the kidneys, serve to maintain phosphate serum concentrations within a typical range of 0.84–1.45 mmol/L in adults. In infants, the range of serum phosphate levels is much higher as compared to adults, *i.e.,* 1.25–2.50 mmol/L, and gradually declines through childhood and adolescence. Overall, less than 1% of the total body phosphate is reported to reside in blood (Vervloet and van Ballegooijen 2018).

In part due to the tight regulatory network, the relationship between phosphate intake and phosphate serum levels is not entirely clear. While several studies suggest that the acute phosphate intake may result in a transient postprandial increase in serum phosphate followed by rapid renal phosphate excretion, evidence for a correlation between chronic dietary phosphate intake and phosphate serum levels is limited.

#### Evidence from epidemiological studies

In a prospective, community-based atherosclerosis risk study (n = 15,732 participants) investigating calcium phosphate levels as a risk factor for coronary heart disease, stroke, and mortality, an association was reported between serum phosphate levels and dietary phosphorus intake over the preceding year, as assessed by an interviewer-administered 66-item food-frequency questionnaire (FFQ) (Foley et al. 2008). However, no details on exposure assessment were provided.

In a cross-sectional analysis, fasting morning serum phosphorus concentrations were measured in a random cohort of 2,664 participants with phosphorus intake being assessed via FFQ (Gutierrez et al. 2012). In multivariable-adjusted analyses, a higher intake of meat or foods enriched with phosphorus additives was not associated with higher serum phosphorus levels, leading the authors to conclude that “excess intake of processed and fast foods may not impact fasting serum phosphorus concentrations among individuals without kidney disease”.

In a cross-sectional study involving 15,513 participants in the Third National Health and Nutrition Examination Survey (NHANES III), dietary intakes of phosphorus and phosphorus-rich foods as assessed by a 24-h dietary recall and a 1-month FFQ were found to only weakly correlate with serum phosphorus concentrations (de Boer et al. 2009). Each increase in dietary phosphorus intake by 500 mg was associated on average with a 0.03 mg/dL (0.1 mmol/L) higher serum phosphorus level after adjustment for age, sex, race/ethnicity, time of day, and fasting status (p < 0.001). No association between dietary phosphorus intake and serum phosphorus levels was observed in a subgroup of participants with a reduced estimated glomerular filtration rate (GFR) (< 60 mL/min/1.73 m^2^). Based on the 1-month FFQ data, consumption of meat (primarily beef) and dairy products was associated with a statistically significant but very minor increase in serum phosphorus concentrations of 0.02 and 0.01 mg/dL/serving/day, respectively, corresponding to 0.06 and 0.03 mmol/L/serving/day in participants without CKD, while other phosphorus-rich foods had no impact on serum phosphorus levels. Depending on the time of day and fasting status, considerable intraindividual variations in serum phosphorus concentrations were observed. Based on these observations, the authors noted that a single measurement of serum phosphorus may not precisely reflect its steady-state concentration. The authors of the study also considered that imprecise exposure estimates based on dietary surveys may introduce bias toward the null association, which may in part explain the weak associations between dietary phosphorus intake and serum phosphate levels.

A cross-sectional analysis of 24-h food recall data from 7,895 adult participants in the National Health and Nutrition Examination Survey (NHANES) 2003–2006, designed to examine the relation between dietary phosphorus intake (both natural organic and inorganic phosphate additives) and serum phosphorus concentration, found an association between serum phosphorus levels and consumption of dairy foods with or without inorganic phosphates and cereals/grains with inorganic phosphates. Dietary phosphorus intake was determined from a 24-h dietary recall on the day of serum sampling. The authors of the study concluded that dairy products and cereals/grains with inorganic phosphate additives lead to a significant increase in serum phosphorus concentration (Moore et al. 2015).

A cross-sectional study in a Japanese cohort of 103 healthy young participants investigated the impact of consumption patterns of rice, bread and noodles on serum phosphate levels (Saito et al. 2021). Dietary habits were collected via a self-administered diet history questionnaire. Serum phosphate levels were higher in individuals that consumed noodles more frequently as compared to the high-bread and high-rice subgroups. Dietary phosphorus was slightly higher in the high-noodle cluster (551 ± 93 vs. 507 ± 87 (bread) and 489 ± 81 mg phosphorus/1,000 kcal (rice)). The authors acknowledge the small study cohort and reporting bias due to dietary exposure assessment via diet history questionnaire as key limitations of their study, calling for further studies to support their findings and assess causality.

#### Evidence from intervention studies

In a controlled phosphate intervention study in 20 young adults with normal renal function, serum phosphate levels were higher in the group receiving a high phosphate diet (regular diet plus 0.55 mM neutral sodium phosphate/kg bw) vs. a group consuming a regular diet supplemented with the phosphate binder lanthanium for 6 weeks (Mohammad et al. 2018).

A 4-week intervention study in thirteen healthy young men consuming either a control diet (1,500 mg phosphate/day), a phosphate supplemented diet (2,300 mg phosphate/day) or a phosphate restricted diet (625 mg phosphate/day) for 9 days revealed increased urinary phosphate excretion in the supplemented group and decreased excretion in the phosphate restricted group, but showed no association between dietary phosphate intake and serum phosphate levels (Antoniucci et al. 2006).

After a 4-day adaptation period on a run-in diet that contained 900 mg of phosphate daily, 66 healthy male and female young adults received either a phosphate-depleted or a phosphate-supplemented diet for 5 days (Burnett et al. 2006). Phosphate depletion was achieved by decreasing the protein content of the diet, resulting in a phosphate intake of 500 mg. Total daily phosphate intake in the phosphate-enriched diet was 2,500 mg (500 mg from the diet and 2,000 mg from the supplements). Fasting blood samples were collected in the morning. While dietary phosphate deprivation resulted in a decrease in serum phosphate levels, dietary phosphate supplementation had no effect on serum phosphate concentrations. Urinary phosphate excretion over 24-h increased by about two-fold in the phosphate-supplemented group, whereas a decrease in 24-h urinary phosphate excretion compared to the run-in period was seen in the phosphate-depleted group.

In a randomized cross-over trial in 10 healthy young men with normal renal function, participants received a standardized diet (1,000 mg phosphorus equivalents/day) supplemented with either a phosphate binder (low P_i_^11^ diet) or phosphate capsules (750 mg phosphorus, high P_i_ diet) for 5 days (Gerber et al. 2025). Blood was collected at the end of each dietary period from overnight fasted participants. High P_i_ intake was associated with higher plasma P_i_ concentrations and a significantly higher 24-h excretion of P_i_ via the kidneys compared to phosphate depletion, whereas no significant differences in renal tubular P_i_ reabsorption were observed.

A double-blind placebo-controlled crossover study with 29 healthy male and female participants investigated the acute effect of excessive dietary phosphorus on the postprandial levels of inorganic phosphate (Volk et al. 2022). Following a 7-day run-in period on a defined dietary protocol, postprandial phosphate serum levels were monitored for up to 8 h after intake of a single dose of 700 mg phosphorus as NaH_2_PO_4_ or a sodium-adjusted placebo with a test meal. Although plasma concentrations in both groups remained within the typical range of 0.84–1.45 mmol/L, significantly higher postprandial phosphate plasma concentrations were observed in the phosphorus group compared to the placebo group throughout the entire observation period. Maximum differences in plasma phosphate concentrations between the phosphorus and the placebo group of  ~ 0.5 mmol/L were observed 3 h after intake. Increased plasma concentrations in the phosphorus group were accompanied by an increased urinary phosphate excretion and a reduced tubular phosphate reabsorption. The authors concluded that despite rapid homeostatic counterreactions that promote renal phosphate excretion, “the acute effects of excessive phosphorus from an inorganic phosphate additive are capable of increasing plasma phosphate concentrations for several hours postmeal”.

In a study by Turner et al., after overnight fasting 18 healthy young adult female and male participants consumed meals containing the same amount of phosphorus but composed of either foods containing phosphorus with lower bioavailability or foods containing inorganic phosphate additives with higher oral bioavailability (Turner et al. 2024). The study followed a randomized cross-over design separated by > 7 days. Blood and urine samples were collected for up to 3 h after the meal. Serum phosphate levels were reported to be higher between 45 min and 3 h after the meal high in inorganic phosphate additives as compared to the low-additive meal. Urinary phosphate excretion was also significantly increased in the high phosphate additive group at 120 and 180 min. In a further study arm, 9 female and 5 male participants were requested to adhere to a 5-day meal plan that reflected the recommended daily allowance for phosphorus (701–742 mg P/day) with or without an additional 400 mg inorganic phosphorus supplement (high phosphate group). Participants were then subjected to an oral phosphate tolerance test, in which they consumed an inorganic phosphate drink containing 500 mg phosphorus (sodium phosphate) within 5 min. Irrespective of the diet conditions, serum phosphate concentrations were increased 1 h after consumption of the inorganic phosphate drink and returned to baseline after 2 h. Urinary phosphate excretion was increased in both groups 2 and 3 h after the phosphate load, but slightly higher in the high phosphate group. However, the absolute change was the same in both diet groups, leading the authors to conclude that both groups showed a similar capacity regarding the acute excretion of phosphate.

Uenishi et al. studied the effects of animal protein and plant protein-rich diets on postprandial phosphorus levels in 12 healthy young men (Uenishi et al. 2024). While the phosphorus content of the animal protein and plant protein-rich diets were equivalent, the animal protein-rich diet, but not the plant-based diet, resulted in a small transient postprandial increase in plasma phosphorus levels 2 h after intake, which returned to pre-meal levels after 4 h. In both groups, urinary phosphorus excretion was increased 2 and 4 h after the meal, but this effect was more pronounced in the animal protein-rich group.

### Evidence from animal studies

In a study in male Sprague–Dawley rats, animals were either fed ad libitum a standard commercial rat chow containing 0.52% phosphate or a low-phosphate diet containing 0.1% phosphate for 7 days (Lee et al. 2017). Under deep anesthesia, the femoral artery, jugular vein, bladder and trachea were cannulated, and a cannula was placed into the duodenum to allow instillation of phosphate (1 mL of 10 mM or 1.3 M KH_2_PO_4_ in buffer) or isosmolar control buffer without phosphate. Serum phosphate levels were measured for up to 45 min after instillation. Instillation of 1.3 M phosphate into the duodenum resulted in a significant increase in plasma phosphate concentrations in rats maintained on a normal-phosphate and a low-phosphate diet. Increased phosphate concentrations in response to the phosphate bolus were accompanied by an increase in plasma PTH concentrations. No changes in serum phosphate levels were observed after application of a more physiological phosphate load of 10 mM KH_2_PO_4_. The authors concluded that “dietary phosphate load can cause postprandial hyperphosphataemia and stimulation of PTH release from parathyroid glands in order to maintain phosphate homeostasis”.

In a study investigating – among others – the effects of dietary inorganic phosphate on serum phosphate levels, rats maintained on a high-phosphate diet (1.2% inorganic phosphate) for 7 days exhibited a moderate increase in serum phosphate concentrations compared to those on a normal (0.8% inorganic phosphate) or low-phosphate (0.1% inorganic phosphate) diet (high: ~ 8 mg/dL, normal: ~ 7.5 mg/dL, low: 5 mg/dL) (Giral et al. 2009). In rats fed a low phosphate diet for 4 h in the morning for 7 consecutive days, an acute switch to a high phosphate diet for 4 h resulted in a rapid and marked increase in postprandial serum phosphate concentrations (up to 16 mg/dL) within 2–4 h.

In healthy cats, supplementation of normal diets with the food additives H_3_PO_4_ and NaH_2_PO_4_ resulted in an increase in postprandial serum phosphate concentrations 3 h after food intake associated with increased renal phosphorus excretion, whereas a slight but statistically significantly decrease in preprandial serum phosphate levels (minimum 12 h after the last meal) was observed in cats supplemented with inorganic phosphate when compared to controls (Steffen and Dobenecker 2023).

Amann et al. investigated the impact of a high (1.2% w/w) vs. low (0.8% w/w) phosphorus diet in sham operated rats and rats after subtotal nephrectomy (Amann et al. 2003). While subtotal nephrectomy had no impact on serum phosphorus levels, higher fasting serum phosphorus concentrations were observed in animals placed on the high phosphorus diet for 8 weeks compared to those maintained on a low phosphorus diet.

In male C57BL/6JRj mice maintained on either a high (1.2% w/w) or a standard (0.6% w/w) phosphate diet for 58 weeks, a small but statistically significant increase in plasma phosphate levels accompanied by an about twofold increase in urinary phosphate excretion was reported in the high-phosphate group (Ugrica et al. 2021).

#### Overall evidence on the correlation between phosphate intake and phosphate levels in serum

Overall, there is evidence from human intervention studies and experimental studies in animals for a transient postprandial rise in serum phosphorus levels following dietary phosphorus intake. The peak in the serum phosphate concentration appears to be rapidly compensated by an increased urinary excretion of phosphate. Individual longer-term studies in animals and human epidemiological studies suggest that dietary intake of phosphorus may cause a small increase in steady-state serum phosphorus levels associated with an increased renal phosphorus excretion. While some authors suggest that a postprandial rise in serum phosphate levels may increase the risk for adverse health effects, the significance of the rather minor effects of dietary phosphorus on serum phosphorus levels remains unclear. Establishing a correlation between dietary phosphorus intake and serum phosphorus levels in human studies is complicated by limitations of dietary exposure assessments methods, limited data on phosphorus levels in various foods, differences in oral bioavailability of phosphorus from different dietary sources, and significant intraindividual variations in serum phosphorus levels that are influenced by the fasting state and circadian rhythm. The latter can only partly be explained by circadian variations of hormonal pathways that regulate phosphate homeostasis (Vervloet et al. 2017). However, the serum phosphorus concentration seems not to be a suitable parameter of the internal exposure to dietary phosphorus.

## Potential toxicity endpoints

EFSA classified phosphates as having low acute oral toxicity and expressed no concerns regarding genotoxicity or carcinogenicity (EFSA, 2019). Additionally, EFSA reviewed the available data on various potential toxicity endpoints, including effects on the kidney, cardiovascular system, bone health and the gastrointestinal tract. In the epidemiological studies reviewed, there were no consistent associations between phosphorus intake via the diet and cardiovascular outcomes. Although some studies suggested a potential association between elevated serum phosphate levels and vascular calcification or arterial stiffness, these studies had limitations (*e.g.*, lack of control for confounding factors), and there was insufficient evidence to establish causality or to define a reference point for cardiovascular effects (EFSA, 2019).

Regarding bone health, limited epidemiological studies have investigated the role of phosphate in the general healthy population (EFSA, 2019). EFSA noted that high phosphate intake, especially in combination with a low calcium intake, can disrupt calcium-phosphate balance, potentially affecting bone mineralization and PTH levels. However, the available data did not show consistent adverse effects on bone mineral density (BMD) or makers of bone turnover in humans at typical exposure levels. Taken together, EFSA concluded that: I) the evidence is insufficient to establish a link between dietary phosphate intake and outcomes relative to cardiovascular health, and II) while high phosphate intake can influence calcium-phosphate regulating hormones, current evidence does not support a clear association between dietary phosphate intake and reduced BMD, or between serum phosphate levels and fracture incidence (EFSA, 2019).

Furthermore, EFSA evaluated the effects of phosphate on the gastrointestinal tract and classified the reported symptoms (*e.g.*, diarrhea, loose stools) as discomfort rather than adverse effects (EFSA, 2019).

Phosphate also plays a role in tooth enamel formation. Although some case reports and small case series have reported some side effects regarding dental health (*e.g.*, effects on tooth roots, pulp chambers and canals, or periapical radiolucency) and suggested that hyperphosphatemia may have contributed to these effects, the underlying mechanism of action remains unclear (Lee et al. 2021). Overall, the available data regarding toxicity endpoints other than the kidney were inconclusive.

Since nephrocalcinosis and/or tubulointerstitial nephropathy have been consistently observed as relevant outcomes of phosphorus/phosphate exposure in animal and human studies and given the insufficient evidence regarding other outcomes (*e.g.*, on bone or cardiovascular effects) in healthy populations, the kidney is considered the primary target organ for phosphate toxicity (EFSA, 2019). This article will therefore focus on the kidney as the most relevant target organ for adverse outcomes associated with excessive phosphorus/phosphate intake. Clinical intervention studies have shown that daily doses of up to 2,000 mg phosphorus (28.6 mg/kg bw/day for a person weighing 70 kg) did not negatively impact kidney function. In contrast, doses of 4,800 mg/day (68.6 mg/kg bw/day for a person weighing 70 kg) were associated with impaired renal function and other adverse effects in the kidney (EFSA, 2019).

The following sections summarize recent evidence linking altered phosphate homeostasis to kidney damage in both animal models and humans.

### Phosphates and kidney damage

#### Excessive phosphorus intake and kidney damage in animal models

The adverse effects of an excessive phosphorus intake in the kidneys have been addressed in several animal species and models. In an early study by Haut et al. (Haut et al. 1980), a dietary phosphate load (0.5–2.0% phosphorus in the diet for 18 weeks) was shown to induce renal tubular damage and interstitial fibrosis in rats, which partially recapitulate lesions observed in the elderly and patients with CKD. Further studies reported that exposure of female rats to high dietary phosphorus amounts (approx. 0.4–1% phosphorus in the diet) for 2–12 weeks led to the development of nephrocalcinosis or calcification of the kidney (Hitchman et al. 1979; Mars et al. 1988; Peterson et al. 1996; Tsuchiya et al. 2004). This kidney disease is responsive to dietary phosphorus and to the dietary calcium:phosphorus ratio (Cockell and Belonje 2004; Cockell et al. 2002; Reeves et al. 1993; Shah and Belonje 1991). An inverse calcium:phosphorus ratio (*i.e.,* less calcium than phosphorus) appears to be the primary risk factor, although the absolute amounts of calcium and phosphorus also influence nephrocalcinosis (Shah and Belonje 1991). Matsuzaki et al. (Matsuzaki et al. 2007) reported that a high-phosphorus diet (1.5% phosphorus in the diet) for 14 days induced the expression of the bone-specific protein osteopontin in the renal tubules and suggested that this increase may be involved in the formation of calcium deposits induced by a high-phosphorus diet, since osteopontin is assumed to interact with hydroxyapatite via γ-carboxyglutamic acid residues (Oldberg et al. 1986). Moreover, Takasugi et al. showed that a high intake of phosphorus (1.2% phosphorus in the diet) synergistically induced nephrocalcinosis in the presence of an estrogenic stimulus and that FGF23 was involved in the induction of nephrocalcinosis triggered by a high phosphorus intake, in part through the fibroblast growth factor 1 receptor (Takasugi et al. 2020). By making use of the remnant-kidney model in rats, Ibels et al*.* reported that renal calcification produced by the altered phosphorus metabolism present in the uremic state incites an inflammatory and fibrotic reaction leading to destruction of the remnant kidney, which can be prevented by dietary phosphate restriction (Ibels et al. 1978).

When normal adult mice were placed on a high-phosphate diet (2% inorganic phosphate in the diet), depositions of collagen and calcium phosphate became detectable within 4 weeks in renal tubules around the cortico-medullary junction, which progressed to overt tubulointerstitial fibrosis and nephrocalcinosis within 8 weeks (Hirano et al. 2020). In mice exposed to an excessive phosphate intake (1–2% inorganic phosphate in the diet) for 12 weeks, circulating levels of FGF23 were increased and led to a suppression of phosphate reabsorption in the renal tubules, which in turn raised the phosphate concentration in the tubule fluid (Shiizaki et al. 2021). Once it exceeded a threshold, microscopic particles containing calcium phosphate crystals appeared in the tubule lumen, which damaged the tubule cells and induced interstitial fibrosis as well as a progressive nephron loss (Shiizaki et al. 2021).

Cats are known to develop clinical signs of CKD with a high prevalence (1–3% in the general feline population and up to 35% in geriatric feline populations) (Greene et al. 2014), CKD being a major cause of death in cats (Grauer 2005). Detrimental effects of nutritional phosphorus excess on renal health have been described in cats (Laflamme et al. 2020). Dobenecker et al. reported that feeding healthy adult cats a diet containing an excessive amount of highly available phosphorus (1.6%) in the diet for 29 days led to glucosuria and microalbuminuria as well as to a decreased creatinine clearance (Dobenecker et al. 2018). Furthermore, adding phosphoric acid and sodium phosphate (total concentration of phosphorus: 13.1 g/kg dry matter) to the diet of healthy cats for 28 days caused a significant increase in serum phosphate, calcium and FGF23 as well as in renal phosphorus excretion (Steffen and Dobenecker 2023). A retrospective study, in which data regarding phosphorus intake in the feeding history of cats (assessed through dietary questionnaire) prior to the diagnosis of CKD was evaluated, revealed that cats with CKD showed significantly higher phosphorus intakes prior to diagnosis than the control cats (Boswald et al. 2018). Increases in serum phosphate, PTH and FGF23 levels have been described as being risk or prognostic factors in the progression of CKD in cats (Chakrabarti et al. 2012; Finch et al. 2013, 2012; Geddes et al. 2015; King et al. 2007). At the present time, it is not clear whether these increases actually contribute to renal injury or simply are markers of ongoing renal injury (Chang and Anderson 2017). In cats with induced renal failure, restriction of dietary phosphorus resulted in less renal mineralization, as well as in a reduction of fibrosis and of inflammatory cell infiltration (Ross et al. 1982).

Chronic kidney disease is common in dogs, with a reported prevalence of up to 25% in dogs presented to a veterinary teaching hospital (Bartlett et al. 2010; Guidi et al. 2012; Lund et al. 1999), and is associated with hyperphosphatemia^12^, decreased vitamin D metabolite concentrations and hyperparathyroidism. In this context, Cortadellas et al. (Cortadellas et al. 2010) showed that hyperparathyroidism and hyperphosphatemia occur frequently in dogs with naturally occurring CKD, even at early stages of the disease in some dogs. Moreover, hyperphosphatemia has been associated with a more rapid progression of CKD and decreased survival in dogs, in which a CKD was experimentally induced (Brown et al. 1991; Finco et al. 1992). In dogs with an induced CKD, dietary phosphorus restriction reduced the associated morbidity and improved survival (Brown et al. 1991; Finco et al. 1992; Slatopolsky et al. 1972).

The currently available data in studies with rats, mice, cats and dogs show that excess phosphorus, especially when provided as highly soluble inorganic phosphate salts, can damage the kidneys. Evidence in rats, cats and dogs indicates that dietary phosphorus restriction may result in an amelioration of CKD symptoms.

#### Excessive phosphorus intake and kidney damage in humans

In humans, chronic kidney disease is defined as a decreased kidney function characterized by a GFR of less than 60 mL/min per 1.73 m^2^, or by markers of kidney damage, or both, of at least 3 months duration, regardless of the underlying cause (Webster et al. 2017). Markers of kidney damage are albuminuria, urinary sediment abnormality, electrolyte or other abnormality due to tubular disorder, histological abnormalities, structural abnormalities detected by imaging and a history of a kidney transplantation. End-stage renal disease (ESRD) is the final, permanent stage of CKD, in which kidney function has declined to the extent that the kidneys can no longer sustain their essential physiological functions independently, and a renal replacement therapy (dialysis or kidney transplantation) becomes necessary.

In a retrospective longitudinal cohort study, Sim et al. reported that higher serum phosphorus levels were associated with a greater risk for ESRD and mortality within a large ethnically diverse population with normal kidney function (Sim et al. 2013). Mehrotra et al*.* analyzed the association between serum phosphorus levels and the risk for progression to ESRD and showed that individuals in the highest quartile for serum phosphorus had a significantly higher risk; however, the risk became non-significant after adjustment for potential confounders (Mehrotra et al. 2013). In the frame of the pSoBid (Psychological, Social, and Biological Determinants of Ill Health) cohort (age of the participants: 35–64 years) (McClelland et al. 2016), analysis of the serum phosphate levels versus the estimated GFR indicated a significant relationship, with a large number of subjects displaying an estimated GFR below 90 mL/min per 1.73 m^2^, indicative of an incipient kidney disease. O'Seaghdha et al. examined whether serum phosphorus levels increased the risk of incident CKD and ESRD in the Framingham Heart Study (mean age of the participants: 42 years) and in the NHANES III (mean age of the participants: 44.3 years), respectively (O'Seaghdha et al. 2011). The serum phosphorus levels in the upper-normal range were associated with a doubling in the risk of developing CKD as well as ESRD (O'Seaghdha et al. 2011). In a cohort with a mean age > 65 years, Schwarz et al. observed that participants with a serum phosphate concentration between 1.23 and 1.39 mM and greater than 1.39 mM had a higher risk to accelerate kidney dysfunction than participants with lower serum phosphate concentrations (< 1.07 mM) (Schwarz et al. 2006). In a *post hoc* analysis of the African American Study of Hypertension and Kidney Disease (AASK), elevated baseline serum phosphorus was independently related to decline in GFR and progression to ESRD (Norris et al. 2006). In a cross-sectional study (mean age of the participants: 70 years), Adeney et al. reported that higher serum phosphate levels, still in the normal range, were associated with a decreased kidney function (Adeney et al. 2009). Chue et al. investigated whether serum phosphate levels correlate with the progression of renal dysfunction in patients with early CKD (mean age of the participants: 59 years) (Chue et al. 2011). The results obtained showed that serum phosphate independently predicts decline in renal function in early CKD (Chue et al. 2011). In a study by Zoccali et al., patients with phosphate levels above the median (mean age of the participants: 49 and 45 years in the third and fourth phosphate quartile, respectively) progressed significantly faster either to ESRD or to a composite endpoint of doubling of serum creatinine and ESRD, when compared to patients with phosphate levels below the median (mean age of the participants: 52 years) (Zoccali et al. 2011). Based on these data, Zoccali et al. concluded that phosphate is an independent risk factor for progression of renal disease among patients with proteinuric CKD (Zoccali et al. 2011). Voormolen et al. analyzed whether plasma phosphate levels were associated with renal function loss and mortality in pre-dialysis patients (Voormolen et al. 2007). The data show that a high plasma phosphate level is an independent risk factor for a more rapid decline in renal function and a higher mortality during the pre-dialysis phase (Voormolen et al. 2007). Taken together, these data suggest that high serum phosphate levels may contribute to renal dysfunction in healthy individuals and may worsen CKD progression (Rubio-Aliaga 2020; Rubio-Aliaga and Krapf 2022). However, these studies are observational in design and therefore cannot be used to define whether serum phosphorus levels are indeed causally related to CKD states. Only randomized interventional studies looking at the effects of a correction of serum phosphorus levels on the progression of kidney disease will be able to clarify the role of serum phosphate in the genesis of renal pathologies, as also proposed by Nadkarni and Uribarri (Nadkarni and Uribarri 2014).

## Prevalence of chronic kidney disease (CKD) in humans

The Global Burden of Diseases, Injuries, and Risk Factors Study, coordinated by the Institute for Health Metrics and Evaluation (University of Washington, Seattle, WA), provides a systematic scientific assessment of published, publicly available as well as contributed data on disease and injury incidence, prevalence, and mortality for an exhaustive list of diseases and injuries (GBD 2020a, 2020b). Due in part to the rise in risk factors, such as obesity and diabetes mellitus, the number of patients suffering from CKD has continued to increase over time, affecting about 700 million individuals worldwide in 2017 (GBD 2020a, 2020b). Further studies have even estimated that up to 850 million patients worldwide were affected by a CKD in 2017 (Jager et al. 2019; Kovesdy 2022). Moreover, CKD has been identified as one of the most prominent causes of death in this century (GBD 2020a, 2020b; Jager et al. 2019; Kovesdy 2022) resulting in 1.2 million deaths in 2017 (GBD 2020a, 2020b; Jager et al. 2019; Kovesdy 2022). An additional 1.4 million deaths from cardiovascular disease were attributable to impaired kidney function, representing 7.6% of deaths due to cardiovascular disease in 2017. Deaths due to CKD and cardiovascular disease deaths attributable to impaired kidney function represented 4.6% of total mortality. Ranked as the 17th leading cause of death in 1990, CKD has increased in importance, ranking as the 12th leading cause of death in 2017 (GBD 2018, 2020a, 2020b; Jager et al. 2019; Kovesdy 2022). As mentioned above, chronic kidney diagnoses resulted in 1.2 million deaths in 2017, a number projected to rise by 2040 to 2.2 million in a best-case scenario and up to 4.0 million in a worst-case scenario (Foreman et al. 2018).

Mortality does not give a complete picture of the burden of disease borne by individuals in different populations. The overall burden of disease is assessed using the disability-adjusted life year (DALY), a time-based measure that combines years of life lost due to premature mortality (YLLs) and years of life lost due to time lived in states of less than full health or years of healthy life lost due to disability (YLDs) (WHO 2020). One DALY represents the loss of the equivalent of one year of full health (WHO 2020). In 2019, the ten leading causes of DALYs in age groups 50–74 years and ≥ 75 years largely overlapped (GBD 2020a). Ischaemic heart disease, stroke, chronic obstructive pulmonary disease (COPD), diabetes, lung cancer, CKD and age-related hearing loss appeared in the top ten in both age groups. For ages 50–74 years, low back pain, cirrhosis, and road injuries were the remaining top-ten-ranking causes of DALYs, whereas Alzheimer’s disease and other dementias, lower respiratory infections and falls appeared in the top ten for those aged 75 years and older. The most notable changes in top ten causes in these two age groups between 1990 and 2019 were large declines in age-standardised DALY rates for ischaemic heart disease, stroke, COPD, cirrhosis and road injuries, but increases in DALY rates for diabetes and CKD (GBD 2020a).

In 2016, Girndt et al*.* reported that approximately 2.3% of the adult German population, aged 18 to 79 years, had an estimated GFR of < 60 mL/min/1.73 m^2^ (Girndt et al. 2016). In the same group, the estimated overall prevalence for elevated urinary albumin excretion was 11.5%, while that for having either a reduced estimated GFR or albuminuria was 12.7%. Taken together, the data show that about 1.53 million adults in the age group 18 to 79 years in Germany in 2011 had a reduced estimated glomerular filtration rate. Furthermore, there was a strong association with age: while kidney damage was very rare in people younger than 50 years, every eighth person aged 70 to 79 years was affected (Girndt et al. 2016). In an earlier study, Schaeffner et al*.* reported that about 30% of the participants aged 70 years or older in the Berlin Initiative study had an estimated GFR of < 60 mL/min/1.73 m^2^ (Schaeffner et al. 2012).

## Exceedance of the acceptable daily intake (ADI) in children

According to EFSA, dietary phosphate exposure exceeds the ADI in healthy infants, toddlers and other children at mean consumption levels, and at the 95th percentile for infants through adolescents (EFSA 2019). While no immediate safety concerns were identified for infants < 16 weeks consuming human milk or formula, the chronic high exposures during childhood remain a matter of concern, since experimental evidence suggests that excessive dietary phosphate can lead to kidney calcification through intraluminal calcium phosphate particles triggering renal inflammation via activation of the toll-like 4 receptor (TLR 4) (Bacchetta et al. 2021; Rubio-Aliaga and Krapf 2022). In line with the scientific opinion of EFSA (EFSA 2019), Cuadrado-Soto et al*.* highlighted that within the Nutritional Study in Spanish Pediatric Population (EsNuPi) involving 1,448 Spanish children (aged 1–10 years), phosphate exposure needs to be addressed, given that all children exceeded the recommended phosphorus intake (Cuadrado-Soto et al. 2020). Moreover, the phosphate intake in children consuming standard milk was significantly higher than in those 1–3 years old children consuming adapted/fortified milk (Cuadrado-Soto et al. 2020).

Several studies reported that hyperphosphatemia is indeed observed in pediatric patients with CKD. In an earlier study of 3,673 infants and children with CKD in the US, hyperphosphatemia became significant as the GFR declined below 50% of its normal function; when GFR fell below 25%, hyperphosphatemia occurred in parallel with hypocalcemia and renal osteodystrophy (Chan et al. 2002). In a cross-sectional study of 73 children aged 2–18 years with CKD in Kazakhstan, hyperphosphatemia was diagnosed in 43.8% of the children, particularly in pre-dialysis and dialysis patients, and serum phosphate levels significantly increased, starting at CKD stage 3 compared to stage 1, with a moderate correlation between phosphate levels and CKD stages^13^ (r = 0.43) (Balmukhanova et al. 2020). In a cross-sectional study of 43 children aged 2–18 years with CKD in Bangladesh, serum phosphate levels increased with disease progression and were identified as an independent predictor of increased carotid intimal-medial thickness, a marker of vascular damage, with a correlation coefficient of r = 0.715, thus suggesting that phosphate levels may contribute to vascular complications in children with CKD (Rahman et al. 2022).

In a retrospective study of 26 children (mean age 12 years; 42% male) with incidentally diagnosed advanced kidney failure in the US, hyperphosphatemia was found in 24 patients (92%) at presentation. Median serum phosphorus levels were significantly higher in the non-glomerular (n = 14, 10 mg/dL) than in the glomerular group (n = 12, 6.9 mg/dL), alongside hyperparathyroidism in 25 patients (96%) (Abukwaik et al. 2022). In a Korean cohort study of 431 children with CKD stages 3–5, the prevalence of hyperphosphatemia significantly increased with advancing CKD stage (17.4%, 23.7%, and 41.2% from stages 3b, 4, and 5, respectively), as did the prevalence of hyperparathyroidism (37.3%, 57.4%, 55.3%, and 52.9% from stages 3a, 3b, 4, and 5, respectively) (Jung et al. 2023). In a retrospective study of 43 children with CKD stages 3–5 not on dialysis in Brazil, 51% had a short stature at baseline, which persisted up to the final follow-up, with PTH levels showing an inverse correlation with the height/length z-scores (r = −0.39, at baseline; r = −0.35, at the final follow-up), and serum phosphate levels above the normal range being observed in more children with short stature than in those with an adequate height (Melo et al. 2024).

Other causes of hyperphosphatemia in children include hypoparathyroidism or thyrotoxicosis that can enhance renal phosphate reabsorption (Khan and Khan 2025). Besides, hyperphosphatemia in children can also be genetically determined. Several genetic deficiencies can lead to hypoparathyroidism, pseudohypoparathyroidism, and reduced FGF23 activity, thereby altering phosphate homeostasis (Lee et al. 2021). Physiologically, binding of FGF23 to FGF receptor-1 (FGFR1) and coreceptor KLOTHO leads to downregulation of sodium/phosphate cotransporters NPT2a and NPT2c (NPT: sodium-dependent phosphate transport protein 2) and 25-hydroxyvitamin D-1α-hydroxylase, converting inactive 25-hydroxyvitamin D to 1,25(OH)2D and finally resulting in inhibition of phosphate reabsorption and active vitamin D formation in renal proximal tubules (Bacchetta et al. 2020; Prie and Friedlander 2010; Shimada et al. 2004).

Several nutritional guidelines emphasize the importance of limiting dietary phosphate intake in children with CKD, as phosphate accumulation in the body begins early during CKD progression and plays a significant role in the development of CKD-related mineral and bone disorders (Dasgupta et al. 2013; Ketteler et al. 2018; McAlister et al. 2020; Panzarino et al. 2022). The main challenge lies in the fact that, although dairy products provide most of the calcium necessary for the bone development of children, they also considerably contribute to phosphate intake. Importantly, inorganic phosphate salts used as food additives have nearly 100% bioavailability when compared to organic phosphate in natural foods, making food products enriched with phosphorus-based food additives particularly concerning regarding their phosphate load (Duong et al. 2022). Current recommendations suggest limiting dietary phosphate intake within the suggested dietary intake (SDI) range in mild–moderate CKD stages, and to the lower limit of SDI in patients with advanced CKD and persistent hyperphosphatemia (McAlister et al. 2020).

There is a considerable amount of experimental and observational evidence connecting high serum phosphate levels to negative effects on kidney function in children with CKD. Whether this is also the case in healthy children remains to be shown. The interpretation of the relationship between hyperphosphatemia and renal dysfunction is complicated by the potential for reverse causality, *i.e.,* the possibility that increased serum phosphate levels are a result of the declining kidney function rather than elevated phosphate being responsible for kidney damage. Most available evidence comes from cross-sectional or retrospective studies, but these types of studies cannot establish a causal relationship, *e.g.*, between dietary phosphate intake and CKD. Only one randomized controlled trial testing magnesium supplementation has been conducted in children with CKD (Pandango et al. 2023), highlighting the critical lack of interventional studies that could help determine causality. The mechanisms linking blood phosphate levels to growth retardation and vascular complications in children with CKD are not yet completely understood, and it remains unclear whether early intervention to control blood phosphate levels could prevent or slow these complications.

## Assessment

Since phosphorus is a nutrient, in contrast to contaminants or residues, both the risks from excessive intake and those from insufficient intake must be considered in the risk assessment of this mineral (Bruins et al. 2015). For this reason, the concept of a safe intake range for minerals, which applies for both minerals and vitamins, was introduced and established internationally as early as the 1990s. For each nutrient, an intake range is defined, which is bounded at the lower end by the respective intake reference value or Recommended Daily Allowance (RDA) and at the upper end by the Tolerable Upper Intake Level (UL) (Weißenborn et al. 2018). The adequate intake of phosphorus for adults is 550 mg/day (EFSA 2015). Exposure surveys such as the German MEAL study (Ptok et al. 2024b) showed that the recommended daily intake for phosphorus is estimated to be achieved by 94% of adults, 91% of children and adolescents as well as 98% of infants and toddlers (Ptok et al. 2024b). The food groups “cereals and cereal products”, “milk and dairy products”, and “meat and meat products” contribute the most to the daily phosphorus intake. The prevalence of inadequate phosphorus intake was found to be very low in the different population groups, similarly to exposure surveys in other European countries (Welch et al. 2009).

Due to a lack of data from epidemiological and human intervention studies, EFSA did not propose an UL for phosphorus in its evaluation of phosphates as a food additive. The SKLM agrees with EFSA that setting a health-based guidance value such as the ADI for phosphate presents unique challenges due to its dual role as an essential nutrient and potentially harmful substance. While clinical data suggest adverse effects at doses above 4,800 mg/day (68.6 mg P/kg bw/day) and a possible NOAEL of 2,000 mg/day (28.6 mg P/kg bw/day) (EFSA 2019), applying traditional safety factors to account for sensitive populations (particularly the estimated 10% with reduced renal function) and for exposure duration would result in an ADI below essential nutritional requirements. For comparison, EFSA’s adequate intake values range from 160–640 mg P/day for different age groups, highlighting the relatively narrow margin between nutritional needs and potential adverse effects. This suggests that a conventional ADI approach based on the available, limited human data may not be appropriate for phosphate risk assessment. Instead, the current ADI of 40 mg P/kg bw/day (corresponding to an acceptable intake of phosphorus of 2,800 mg/day for a 70 kg adult), while based on older animal studies, appears to strike a reasonable balance between ensuring adequate nutrition and protecting against adverse effects in the general population. This ADI was derived from a chronic rat study (Hodge 1960) and a 90-day rat study according to OECD guidelines and the determination of the content of phosphorus in the diet (Seo et al. 2011). In addition, several animal studies have confirmed that the kidney is the main organ affected by excess phosphorus; excess phosphate intake causes increased bone demineralization and calcium release, leading to renal calcification and tubular nephropathy (EFSA 2019). The renal effects are seen after only a few weeks of treatment in experimental animals. A comparison of the histopathological changes in animal studies and those in human kidney samples suggests that the mechanisms described in animal studies are also relevant for humans (EFSA 2019). However, the ADI derived from animal studies may not be sufficiently protective for vulnerable groups such as individuals with impaired renal function. Therefore, targeted risk management measures focusing on this vulnerable population may be more appropriate than establishing a lower general ADI.

Analysis of large human cohort studies (EFSA 2019) showed evidence for an association between serum phosphorus and the incidence of chronic vascular disease (CVD) and some evidence for an association between serum phosphorus and cardiovascular mortality (Dhingra et al. 2007; Hayward et al. 2017). In addition, long-term clinical intervention studies have shown that daily doses of up to 2,000 mg of phosphorus (28.6 mg P/kg bw/day) administered for several months up to 2 years were tolerated without renal impairment, whereas doses of 4,800 mg/day (68.6 mg P/kg bw/day) and higher caused renal impairment (EFSA 2019). These studies suggest that concentrations above the ADI may increase renal health risks, particularly in vulnerable groups such as infants, toddlers and children or individuals with reduced renal function.

Furthermore, based on the currently available exposure assessments, children frequently exceed the existing ADI. EFSA reported that maximum dietary exposure to phosphates (*e.g.,* E338–341, E343, E450–452) from their use as food additives in infants (> 12 weeks to < 12 months), toddlers (≥ 12 to < 36 months), and other children (≥ 36 months to < 10 years) in the brand-loyal scenario is close to the ADI at the mean consumption level and exceeds the ADI at the 95th percentile (Fig. 4). Considering phosphate intake from both natural sources and food additives, the ADI was already exceeded at the mean level for infants, toddlers and other children, and aditionally for adolescents at the 95th percentile (EFSA 2019). The German MEAL total diet study, which included all phosphate sources, also showed that in Germany these young population groups – infants, toddlers and other children – exceed the ADI (Fig. 5), with 51% of infants and 60% of toddlers surpassing this HBGV (Ptok et al. 2024b). In addition to the natural occurrence of phosphorus, the use of phosphorus as a food additive has a major impact on exposure to phosphorus, especially in these young population groups. 

Of note, the exposure estimates presented in this assessment are subjected to some uncertainties. These include some assumptions made by EFSA in its exposure assessment, *e.g.*, conservative assumptions in the brand-loyal exposure scenario that might represent an overestimation of the phosphate intake from its use as a food additive (see also Chapter 4 – Exposure). Furthermore, when no actual use data for food additives in certain food categories were available, EFSA used the maximum permitted levels (MPLs), which could also contribute to a conservative exposure estimate. Although the exposure scenarios taking only into account the use of phosphate as a food additive can be considered conservative and, thus, potentially overestimate the phosphate intake, there is considerable evidence from the EFSA estimates and from the BfR-MEAL Study that the overall dietary phosphorus intake in younger population groups is above the ADI. EFSA considered that these overall dietary exposure estimates based on analytical data and, thus, reflecting the levels of phosphorus in foods independently of their origin should reflect more closely the total phosphorus ingested through the diet. Additionally, EFSA used the middle-bound (MB) approach in its refined exposure assessment scenario, which may have resulted in either an overestimation or an underestimation of the exposure.

A further uncertainty to be mentioned in the exposure assessment is that in the case of the overall exposure to phosphate via the diet, reported use levels were considered whenever analytical data were not available for a certain food category. Additionally, consumption data was often based on food consumption surveys covering only a few days, probably also resulting in an overestimation of dietary intake. On the other hand, some food categories (processed eggs, salts and some alcoholic beverages) were not included, as no data were available, thereby possibly contributing to a slight underestimation of the intake in the general population.

Another uncertainty arises from the conversion factor used to convert other phosphate species into P_2_O_5._ As some of the data submitted to EFSA were not expressed as P_2_O_5_, different conversion factors (based on the E-number of the specific food additive) were used. Furthermore, the food composition databases (*e.g.*, *EFSA Comprehensive European Food Consumption Database)* may lack details about phosphate-containing additives or fail to reflect market variability. Further details on uncertainties relative to the exposure assessment are provided in (EFSA, 2019).

Uncertainties in exposure estimates based on data from the BfR-MEAL Study arise, for example, from the use of older consumption data for adolescents, adults, and the elderly. These data were obtained from the German National Nutrition Survey II (*Nationale Verzehrsstudie II*, NVS II), conducted in 2005/2006, and therefore might not reflect recent changes in dietary patterns/consumption trends in these population groups (*e.g.*, increased consumption of plant-based alternatives). However, more recent consumption data are available for younger population groups: for children aged 6 months to 5 years, data were collected between 2014 and 2017 (KiESEL), whereas for children between 6 and 11 years old, data were collected between 2015 and 2017 (EsKiMo II). These more recent datasets provide a more accurate information on current consumption patterns in these younger populations. Additionally, specific consumer groups with distinct dietary patterns, such as vegetarians, are also not adequately represented in the BfR-MEAL data. Moreover, the food list used in the BfR-MEAL Study covers at least 90%, but less than 100%, of the total food consumption, potentially leading to an underestimation of dietary intake.

The ADI defines the safe amount of a substance that a person can ingest over a lifetime without any appreciable risk to health, but does not apply to individuals with a moderate to severe renal impairment. Exceeding the ADI for a short period of time (days) may not pose a health risk to the consumer because the ADI is defined on the basis of a daily lifelong exposure. However, continuous and prolonged exceedance of the ADI should be avoided to prevent long-term adverse health effects.

The precision of the NO(A)EL, the influence of kinetics and the duration of exposure are important factors in determining possible toxicity in the range of the ADI (Renwick and Walker 1993). EFSA concluded that the data from animal studies were sufficiently robust to estimate a NOAEL (EFSA 2019). The NOAEL for phosphorus was identified in both a chronic and a subchronic study in rats (Hodge 1960; Seo et al. 2011) and set by EFSA to 167 mg P/kg bw/day (76 mg/kg bw/day plus a background dietary exposure to phosphorus of 91 mg/kg bw/day). In animal studies, renal effects are observed after weeks of treatment in a dose-dependent manner and the mode of action is similar to that in humans. Therefore, it is likely that exceeding the ADI over a long time could be of concern.

As infants, toddlers and children can be considered as particularly vulnerable groups, the ADI should not be exceeded on a permanent basis. In addition, even though dietary intake of phosphate does not exceed the ADI in adults and the elderly, it is important to consider that about 10% of the adult population may suffer from an undiagnosed form of CKD with reduced renal function and may thus be more vulnerable to phosphorus. As a consequence, the risk of kidney toxicity and CVD may increase. Therefore, there is a need not only to reduce dietary phosphate exposure to levels below the ADI, but also to raise awareness of potential health concerns in vulnerable individuals and the need for risk management measures to mitigate these potential health risks.

## Data gaps and research needs

- While studies like BfR-MEAL provide analytical data for total phosphorus content, more detailed analyses are needed to determine the relative contribution of different phosphate forms (natural vs. additives) in food products. This information would help to better define exposure from different sources, which is of considerable importance in light of the varying bioavailabilities of different phosphate species.
- Reliable data on human oral bioavailability of phosphate species from natural phosphate sources and additives are needed, taking into account the impact of age and disease as well as potential interactions with other dietary components.
- There is a need for more detailed exposure assessment for specific scenarios, particularly for food supplement consumers and formula-fed infants that frequently exceed current guidance values, including regional variations in phosphate consumption patterns across Europe.
- Studies are needed on how improved food labeling (*e.g.,* declaring naturally high phosphorus content, and/or specifying the amount of phosphate from food additives) could help vulnerable populations to manage their intake.
- Long-term studies investigating the correlation between phosphate intake and serum phosphate levels are needed.
- Epidemiological studies that investigate the association between high phosphate intake and risk of decreased cardiovascular function a) with normal renal function and b) with decreased renal function are needed.
- Randomized interventional studies are needed to investigate whether adjusting serum phosphate levels affects the progression of kidney disease, in order to clarify the role of serum phosphate in the development of renal pathologies.
- Given the exceedance of the ADI in children, improved methods to assess and monitor dietary phosphate intake, particularly from food additives, in the pediatric population need to be developed. Additionally, the following types of studies may help better understand the potential health risks of excess phosphate intake:
  ● Interventional studies focusing on CKD risk in children as well as properly designed prospective observational studies in children.
  ● Early intervention studies in at-risk children to determine if phosphate control can prevent later health complications.

## Conclusion

When assessing phosphate intake among consumers in Germany, it is important to distinguish between healthy individuals and those with kidney disease. In the case of the healthy population, the supply of phosphate is adequate, with the recommended AI being achieved by over 90–95% of the individuals (Ptok et al. 2024b). However, a significant proportion of the population in many European countries chronically consumes at least twice the recommended amount of phosphate, leading to an exceedance of the accepted EFSA-derived ADI (40 mg/kg bw/day) in younger population groups, such as infants, toddlers, children, and some adolescents. Evaluation of the experimental studies published up to date shows that chronic high exposure to phosphates may lead to hyperphosphatemia, which in turn results in hyperphosphaturia^14^ as the homeostatic response. Hyperphosphaturia has been shown to cause inflammation of the renal tubules due to the formation of calcium phosphate microparticles. This may lead to a decline in renal phosphate excretion and impaired kidney function. Chronically high phosphate serum levels are associated with a higher incidence of kidney diseases as well as of CVD and a higher mortality rate. Reduced kidney function in patients with chronic renal impairment, which account for roughly 10% of the population in Germany (DGfN 2023), also leads to an increase in blood phosphate levels. Whether these increased blood phosphate levels initiate a vicious circle aggravating the clinical picture of the patients remains to be shown. Nevertheless, patients with impaired kidney function are advised to limit or avoid foods containing high amounts of phosphates, whenever possible, to lower their daily phosphate intake (see *e.g.,* (DGfN 2023; NKF 2024)).

The essential nutrient phosphate is sufficiently supplied through various foods such as milk, cheese, yoghurt, meat, legumes and nuts. However, consumers of food products containing phosphate additives are exposed to significantly higher phosphate quantities due to the higher bioavailability of inorganic phosphate used as additives. Phosphates used as food additives in many food and beverage items contribute significantly to a high phosphate intake. Dietary phosphate exposure from food additives alone already exceeds the ADI in infants, toddlers and other children at the 95th percentile in the brand-loyal, but not in the non-brand-loyal exposure scenario. Exposure to phosphate from all dietary sources exceeds the ADI in population groups such as infants, toddlers and other children at the mean level, and for infants, toddlers, children and adolescents at the 95th percentile (EFSA 2019). The data from the recent German BfR-MEAL Study are generally consistent with this outcome (Ptok et al. 2024b).

## Recommendations and risk management options

Taken together, a restrictive use of phosphate as a food constituent should be viewed as a relevant public health issue. Therefore, the SKLM recommends developing a strategy to reassess the addition of phosphate to foodstuffs, particularly in light of ADI exceedance in susceptible population groups such as infants, toddlers and children already at mean exposure estimates (EFSA 2019; Ptok et al. 2024a, 2024b), as well as to ensure the protection of vulnerable population groups, particularly patients with renal disease.

This strategy includes risk management recommendations such as:

- quantitative and comprehensive labeling of phosphate content in food.
- reconsideration of the food groups for which phosphate addition is approved.
- lowering the maximum levels of phosphate additives in those food categories where their use is permitted.
- forwarding strong recommendations to the food industry to substitute or at least reduce the use of inorganic phosphates in food products and beverages.
- improving risk communication to increase awareness of high phosphate exposure, particularly in young population groups, and develop potential strategies to minimize phosphate intake.

## Supplementary Information

Below is the link to the electronic supplementary material.

Supplementary file1 (PDF 120 kb)

Supplementary file2 (XLSX 69 kb)

Supplementary file3 (PDF 187 kb)

Supplementary file4 (PDF 353 kb)

## Data Availability

The data underlying this article are available in the cited publications and the online supplementary material.

## Footnotes

1 Classification of age groups according to EFSA.

2 Children at a preschool or elementary school age.

3 MEAL Study: Meals for Exposure Assessment and Analysis of Foods (*Mahlzeiten für die Expositionsschätzung und Analytik von Lebensmitteln*).

4 http://www.gnpd.com (last accessed on 19.02.2024).

5 No data were provided by Mintel for certain products, in which phosphates are authorized. Hence, subcategories, in which there is no product labeled with at least one additive, were not included.

6 “Children’s Nutrition Survey to Record Food Consumption” (*Kinder-Ernährungsstudie zur Erfassung des Lebensmittelverzehrs*, KiESEL).

7 “Eating Study as a KiGGS Module” (*Ernährungsstudie als KiGGS-Modul*, EsKiMo II), within the “German Health Interview and Examination Survey for Children and Adolescents” (*Studie zur Gesundheit von Kindern und Jugendlichen in Deutschland*, KiGGS Wave 2).

8 “German National Nutrition Survey II” (*Nationale Verzehrsstudie II*, NVS II).

9 Total levels of phosphorus for infant formula (minimum–maximum): 25–90 mg/100 kcal. Maximum level for infant formula based on soy: 100 mg/100 kcal. (Commission Delegated Regulation (EU) 2016/127, Commission Delegated Regulation (EU) 2016/128, Commission Directive 2006/141/EC, Commission Directive 1999/21/EC).

10 Assuming a density of 1.03 g/mL for human milk. Source: Institute of Medicine (US) Committee on Nutritional Status During Pregnancy and Lactation. Nutrition During Lactation. Washington (DC): National Academies Press (US); 1991. 5, Milk Volume. Available from: https://www.ncbi.nlm.nih.gov/books/NBK235589/ (last accessed on 07.10.2025).

11 P_i_ in this paper refers to phosphorus mass content in all species of phosphate.

12 Abnormally high level of phosphate in blood (typically defined as serum phosphate concentration above 1.45 mmol/L or  > 4.5 mg/dL).

13 CKD stages as defined by the Clinical Practice Guideline for the Evaluation and Management of Chronic Kidney Disease, based on the glomerular filtration rate (GFR) (https://kdigo.org/wp-content/uploads/2017/02/KDIGO_2012_CKD_GL.pdf): Stage 1: normal or high kidney function, GFR ≥ 90 mL/min/1.73 m^2^, but evidence of kidney damage such as hematuria or proteinuria. Stage 2: mildly decreased kidney function, GFR 60–89 mL/min/1.73 m^2^, with other signs of kidney damage. Stage 3a: mildly to moderately decreased kidney function, GFR 45–59 mL/min/1.73 m^2^. Stage 3b: moderately to severely decreased kidney function, GFR 30–44 mL/min/1.73 m^2^. Stage 4: severely decreased kidney function, GFR 15–29 mL/min/1.73 m^2^. Stage 5: kidney failure (End-Stage Renal Disease –ESRD–), GFR < 15 mL/min/1.73 m^2^.

14 Abnormally high level of phosphate in the urine.
