# Supplementary material for: Dietary phosphate exposure–strategies to protect vulnerable population groups
Source: Arch Toxicol. 2026 Feb 7;100(5):1657–84. doi: 10.1007/s00204-025-04274-y (PMC13086773; doi:10.1007/s00204-025-04274-y)
Supplement: Supplementary file 1 — Supplementary file1 (PDF 120 kb) [file 204_2025_4274_MOESM1_ESM.pdf]

Electronic Supplementary Material Table S1

Archives of Toxicology

Dietary phosphate exposure - strategies to protect vulnerable population groups

Alfonso Lampen, Dirk W. Lachenmeier, Patrick Diel, Regina Ensenauer, Lara Frommherz, Sabine Guth, Hans-Ulrich Humpf, Sabine E. Kulling, María A. Villar-Fernández, Wim Wätjen, Angela Mally, Pablo Steinberg

Corresponding author:

Alfonso Lampen. University of Veterinary Medicine Hannover, Institute for Food Quality and Food Safety, Bischofsholer Damm 15, 30173, Hannover, Germany.

E-Mail: alfonso.lampen@bfr.bund.de

Electronic Supplementary Material Table S1. Summary of maximum levels for phosphates, E338-E341, E343, E450-452 (phosphoric acid, Na-, K-, Ca-, Mg-phosphates, di-, tri-, and polyphosphates), across various food categories based on Regulation (EC) No 1333/2008 (EC 2008).

**Food Category**	**Maximum Level expressed as P_2O_5 (mg/kg or mg/L)**
Beverage whiteners for vending machines	50,000
Beverage whiteners, Vegetable oil pan spray	30,000
Processed cheese, Bread and rolls, Fine bakery wares, Vegetable protein drinks, self-raising flour	20,000
Batters	12,000
All categories of foods in dried powdered form except foods for infants and young children, except where specifically provided for	10,000
Liquid egg, Salt and salt substitutes	10,000
Dry powdered dessert mixes	7,000
Whipped cream products, Spreadable fats, Spreadable nut fats, Processed potato products, Sugar confectionery, Decorations, coating, fillings, Breakfast cereals, Meat preparations, Non-heat treated meat products, Heat-treated meat products, Frozen and deep-frozen fish fillets, Frozen and deep-frozen mollusks and crustaceans, Some salted fishes, Sauces, Dietary foods for special medical purposes, Some snacks, Processed nuts	5,000
Glazings for vegetable products, Glazings for meat, Whey protein sport drinks	4,000
Flavored fermented milk products, toppings, Soups and broths, Desserts	3,000
Cereals and cereal products, Dried milk and dried skimmed milk, Flours	2,500
Unripened cheese except mozzarella, Soured cream butter, Noodles, Chocolate and malt dairy-based drinks, Coffee-based drinks for vending machines, instant tea	2,000
Partly dehydrated milk with more than 28% solids	1,500
Dairy products and analogues, Partly dehydrated milk with less than 28% solids, Edible ices, Seaweed based fish roe analogues, Canned crustacean products, surimi, Infant and follow-on formulae, baby foods and foods for young children with several restrictions (only for cereals and fruit-based desserts, only some specific phosphates), Cider and perry, Fruit wine and made wine, Mead, Aromatized wine and wine-based drinks and cocktails, Spirit drinks, certain other alcoholic beverages	1,000
Fruit preparations, candied fruit	800
Flavored drinks	700
Prepared table waters, Sport drinks	500
Chewing gum, Table-top sweeteners, Food supplements in solid form excluding supplements for infants and young children	*Quantum satis*

Quantum Satis: no maximum numerical level is specified, and substances shall be used in accordance with good manufacturing practice, at a level not higher than is necessary to achieve the intended purpose and provided the consumer is not misled.
