# Supplementary material for: Dietary phosphate exposure–strategies to protect vulnerable population groups
Source: Arch Toxicol. 2026 Feb 7;100(5):1657–84. doi: 10.1007/s00204-025-04274-y (PMC13086773; doi:10.1007/s00204-025-04274-y)

Electronic Supplementary Material S3

Archives of Toxicology

Dietary phosphate exposure - strategies to protect vulnerable population groups

Alfonso Lampen, Dirk W. Lachenmeier, Patrick Diel, Regina Ensenauer, Lara Frommherz, Sabine Guth, Hans-Ulrich Humpf, Sabine E. Kulling, María A. Villar-Fernández, Wim Wätjen, Angela Mally, Pablo Steinberg

Corresponding author:

Alfonso Lampen. University of Veterinary Medicine Hannover, Institute for Food Quality and Food Safety, Bischofsholer Damm 15, 30173, Hannover, Germany.

E-Mail: alfonso.lampen@bfr.bund.de

Electronic Supplementary Material ESM3: Dietary exposure to phosphorus (P) in infants < 16 weeks (A), infants (12 weeks–11 months) (B), toddlers (C) and other children (D) (minimum-maximum mean and 95th percentile (P95) across the dietary surveys expressed in mg P/kg bw/day) as estimated by EFSA (2019). Three different exposure scenarios are represented: total phosphorus intake from the diet (yellow) and intake from the use of phosphate as food additive, considering a refined estimated exposure assessment scenario (non-brand-loyal (blue) and brand-loyal (green)). The red line indicates the acceptable daily intake (ADI) of 40 mg P/kg bw/day. FA: food additive.

Source: EFSA FAF Panel, Younes M et al. 2019. Scientific Opinion on the re-evaluation of phosphoric acid–phosphates – di-, tri- and polyphosphates (E 338–341, E 343, E 450–452) as food additives and the safety of proposed extension of use. EFSA Journal 2019;17 (6):5674, 156 pp. https://doi.org/10.2903/j.efsa.2019.5674 (Tables 5b and 10b).

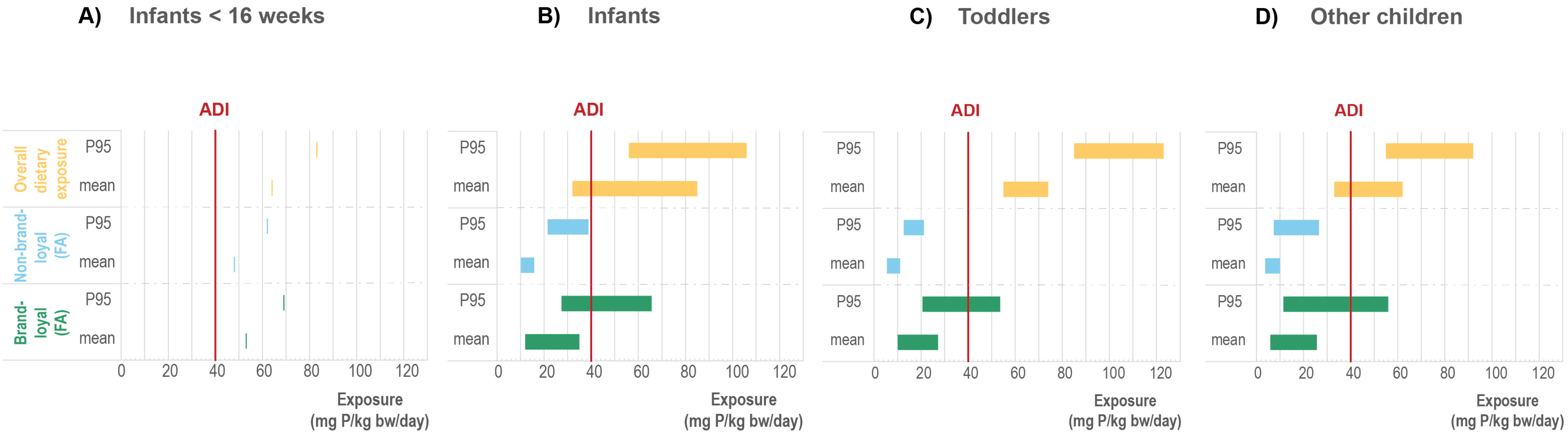

Supplement: Supplementary file 3 — Supplementary file3 (PDF 187 kb) [file 204_2025_4274_MOESM3_ESM.pdf]
