# Supplementary material for: Dietary phosphate exposure–strategies to protect vulnerable population groups
Source: Arch Toxicol. 2026 Feb 7;100(5):1657–84. doi: 10.1007/s00204-025-04274-y (PMC13086773; doi:10.1007/s00204-025-04274-y)

Electronic Supplementary Material S4

Archives of Toxicology

Dietary phosphate exposure - strategies to protect vulnerable population groups

Alfonso Lampen, Dirk W. Lachenmeier, Patrick Diel, Regina Ensenauer, Lara Frommherz, Sabine Guth, Hans-Ulrich Humpf, Sabine E. Kulling, María A. Villar-Fernández, Wim Wätjen, Angela Mally, Pablo Steinberg

Corresponding author:

Alfonso Lampen. University of Veterinary Medicine Hannover, Institute for Food Quality and Food Safety, Bischofsholer Damm 15, 30173, Hannover, Germany.

E-Mail: alfonso.lampen@bfr.bund.de

Electronic Supplementary Material S4: Dietary exposure to phosphates (E338–341, E343, E450–452) from their uses as food additives for food supplements consumers only, in children, adolescents, adults and the elderly (A) and from their uses in foods for special medical purposes (FSMP) consumers only in infants < 16 weeks (infants < 16 w), infants between 12 weeks and 11 months (infants (12 w-11 mos)) and toddlers (B) (minimum–maximum mean and 95th percentile (P95) across the dietary surveys, expressed as mg phosphorus (P)/kg bw per day) as estimated by EFSA (2019). Two different exposure scenarios are represented: total phosphorus intake from the diet (dark grey) and intake from the use of phosphate as food additive (light grey). The red line indicates the acceptable daily intake (ADI) of 40 mg P/kg bw/day. W: weeks; mos: months.

Source: EFSA FAF Panel, Younes M et al. 2019. Scientific Opinion on the re-evaluation of phosphoric acid–phosphates – di-, tri- and polyphosphates (E 338–341, E 343, E 450–452) as food additives and the safety of proposed extension of use. EFSA Journal 2019;17 (6):5674, 156 pp. https://doi.org/10.2903/j.efsa.2019.5674 (Tables 11, 12 and 6 and 7).

A) **Dietary exposure to phosphorus for consumers of food supplements**

B) **Dietary exposure to phosphorus for consumers of FSMPs**

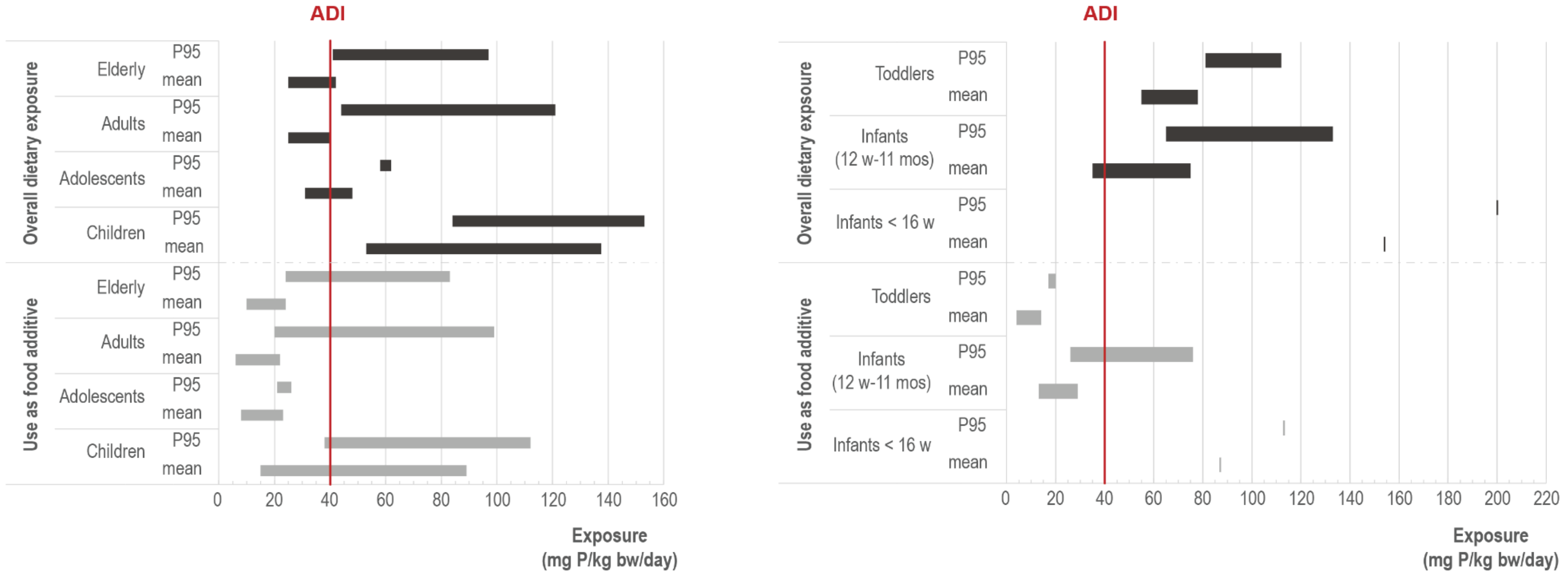

Supplement: Supplementary file 4 — Supplementary file4 (PDF 353 kb) [file 204_2025_4274_MOESM4_ESM.pdf]
